# FtsHi4 Is Essential for Embryogenesis Due to Its Influence on Chloroplast Development in *Arabidopsis*

**DOI:** 10.1371/journal.pone.0099741

**Published:** 2014-06-25

**Authors:** Xiaoduo Lu, Dongyuan Zhang, Shipeng Li, Yanping Su, Qiuju Liang, Hongyan Meng, Songdong Shen, Yunliu Fan, Chunming Liu, Chunyi Zhang

**Affiliations:** 1 Department of Life Sciences, Qilu Normal University, Jinan, China; 2 Qingdao Institute of Bioenergy and Bioprocess Technology, Chinese Academy of Sciences, Qingdao, China; 3 School of Biology and Basic Medical Sciences, Soochow University, Suzhou, China; 4 Biotechnology Research Institute, Chinese Academy of Agricultural Sciences, Beijing, China; 5 National Key Facility for Crop Gene Resources and Genetic Improvement (NFCRI), Beijing, China; 6 Institute of Botany, Chinese Academy of Sciences, Beijing, China; Wuhan University, China

## Abstract

Chloroplast formation is associated with embryo development and seedling growth. However, the relationship between chloroplast differentiation and embryo development remains unclear. Five *FtsHi* genes that encode proteins with high similarity to FtsH proteins, but lack Zn^2+^-binding motifs, are present in the *Arabidopsis* genome. In this study, we showed that T-DNA insertion mutations in the *Arabidopsis FtsHi4* gene resulted in embryo arrest at the globular-to-heart–shaped transition stage. Transmission electron microscopic analyses revealed abnormal plastid differentiation with a severe defect in thylakoid formation in the mutant embryos. Immunocytological studies demonstrated that FtsHi4 localized in chloroplasts as a thylakoid membrane-associated protein, supporting its essential role in thylakoid membrane formation. We further showed that FtsHi4 forms protein complexes, and that there was a significant reduction in the accumulation of D2 and PsbO (two photosystem II proteins) in mutant ovules. The role of FtsHi4 in chloroplast development was confirmed using an RNA-interfering approach. Additionally, mutations in other *FtsHi* genes including *FtsHi1*, *FtsHi2*, and *FtsHi5* caused phenotypic abnormalities similar to *ftshi4* with respect to plastid differentiation during embryogenesis. Taken together, our data suggest that FtsHi4, together with FtsHi1, FtsHi2, and FtsHi5 are essential for chloroplast development in *Arabidopsis*.

## Introduction

Angiosperm embryo and endosperm initiate from double fertilization, where one of the two sperm cells fuse with an egg cell and the other fertilizes with the central cell, respectively [1]. After fertilization, the elongated zygote undergoes an asymmetric cell division to produce a smaller apical and larger basal cell [2]. The apical cell undergoes one-cell, two-cell, four-cell, eight-cell, globular, heart-shaped, torpedo, and bent cotyledon-shaped embryo stages to refine embryonic patterns [3].

The chloroplast, which is an organelle derived from cyanobacteria through endosymbiosis, is a specific type of plastid. Chloroplasts are propagated from a pre-existing plastid through divisions, and formation is initiated from an undifferentiated plastid type; the proplastids. After responding to light, proplastids develop grana, which are stacks of thylakoid membranes for light harvesting, electron transfer, and ATP synthesis. The chloroplast is responsible not only for photosynthesis, but for synthesis and storage of metabolic products (e.g., fatty acids, amino acids, starch) [2], [4].

Chloroplast formation is associated with embryo development and seedling growth. The early differentiation of chloroplasts occurs at the globular-to-heart transition stage during embryogenesis [2], and embryos begin accumulating chlorophyll during the heart-shaped stage. Many developmental and metabolic events occur at the globular-to-heart transition stage. For example, the embryo establishes bilateral symmetry with the emergence of cotyledons [5]. Approximately 40% of embryo-defective mutants are arrested at the globular-heart transition stage [6]. Null mutation of *TIC110*, which functions in the formation of the chloroplast inner envelope translocation channel, leads to embryo lethality [7]. Two ankyrin repeat-containing proteins, EMB506 and AKRP, which are essential for plastid differentiation, affect embryo transition from the globular to heart-shaped stage [8]. Disrupted plastid Nap7, which likely affects Fe-S biogenesis, leads to embryo arrest at the late globular stage [9]. The type I MADS-box gene *AGL23* and the pentatricopeptide repeat protein DELAYED GREENING1 (DG1) are also involved in chloroplast biogenesis during embryogenesis [10], [11]. EMB1303, a chloroplast-localized protein, is essential for chloroplast development. Mutants of *emb1303* show delayed embryo development and severe dwarf and albino seedlings with arrested plastid development at the early stage [12]. In addition, EMB1211, a plastid MORN-containing protein, is essential for the transition from the globular to the heart-shaped stage during embryo development [13]. These observations indicate that normal chloroplast development is required for nourishment, and is an important biological process for normal embryogenesis. It has also been proposed that developing chloroplasts release a signal required for regulating nuclear gene expression, which consequentially affects embryo development [14]–[16].

In contrast, mutations in photosynthesis-related genes do not necessarily cause embryo lethality, and often produce homozygous albino seeds that are morphologically normal. Such seeds can typically germinate and grow to various extents on sugar-rich medium, resulting in albino, de-pigmented, pale green to yellow seedlings or variegated seedlings [6], [17], [18]. The majority of plastidic proteins essential for the globular-heart transition are involved in the transcriptional and translational machineries of the plastids [19]. Interestingly, some chloroplast-encoded genes are essential for cell viability [20]. Disruption of the housekeeping chloroplast function often results in embryo lethality, yet rarely in gametophyte lethality [21], [22].

Proteases play crucial roles in the biogenesis and maintenance of chloroplasts. To date, four protease families have been identified in chloroplasts: Clp, FtsH, Lon, and Deg. However, only one of the ClpPR protease complexes, ClpP5, is known to be essential for the transition from the globular to the heart-shaped stage during embryo development [23]. Filamentation temperature-sensitive H (FtsH) is an ATP-dependent metalloprotease that controls plastid protein quality. There are 12 nuclear-encoded *FtsH* genes in the *Arabidopsis* genome [24] and four potential FtsH proteases in *Synechocystis* [25]. Nine of the *Arabidopsis* FtsHs are targeted to chloroplasts and three are targeted to mitochondria [26]. In *Synechocystis*, a hetero-oligomeric complex composed of FtsH2 and FtsH3 functions in removing damaged D1 protein [27]. In *Arabidopsis*, the thylakoid FtsH protease possesses proteolytic activity and is involved in the turnover of the D1 protein in the photosystem II (PSII) reaction center within the context of repair from photoinhibition [28], [29] and degradation of unassembled proteins [30]. Chloroplast-targeted FtsH2 and FtsH8, FtsH1 and FtsH5, and FtsH7 and FtsH9 are closely related pairs. Inactivation of FtsH1 and FtsH5 or FtsH2 and FtsH8 results in a heterotrophic albino phenotype [31].

There are five nuclear-encoded *FtsHi* genes (FtsHi1 to FtsHi5, and the “i” indicates proteolytic inactivation [32]) in the *Arabidopsis* genome that display a high degree of similarity to FtsHs at the protein level. However, they lack a Zn-binding site required for proteolytic activity [32], [33]. FtsHi1 is required for chloroplast development [34]. However, how other FtsHi proteins affect embryo development remains unknown.

In this study, we used a reverse-genetics approach to explore the function of the *FtsHi* genes using *Arabidopsis* T-DNA insertion mutants. Mutations of the *FtsHi4* gene (At5g64580) led to embryo lethality and failed thylakoid formation, similar to other *ftshi* mutants including mutants of *FtsHi1* (At4g23940), *FtsHi2* (At3g16290), and *FtsHi5* (At3g04340). FtsHi4 was localized in chloroplasts as a thylakoid membrane-associated protein. A significant decrease in D2 and PsbO protein accumulation occurred in the homozygous *ftshi4* mutant embryos. Moreover, we demonstrated that knock-down of FtsHi4 expression using an RNA interfering approach resulted in defects in PSII functioning. These results indicated that FtsHi4 is required for PSII formation during embryogenesis. Taken together, our data suggest that FtsHi4, together with other FtsHi proteins, are essential for plastid development during embryogenesis in *Arabidopsis*.

## Materials and Methods

### Plant material and growth conditions

The *Arabidopsis thaliana* ecotype Columbia-0 was used as the wild-type. The *ftshi4-1* mutant allele was isolated from a population of transgenic plants generated in our laboratory that displayed white ovules. Mutant seeds obtained from the *Arabidopsis* Biological Resource Center (ABRC; The Ohio State University) were as follows: *ftshi4-2/emb3144* (Salk_113657), *ftshi3-1* (GK_723C06), *ftshi3-2* (GK_555D09), *ftshi1-1*/*emb2458* (CS16181), *ftshi2-1*/*emb2083-4* (CS16209), *ftshi2-2/emb2083-3* (CS16208), *ftshi2-3/emb2083-2* (CS16167), and *ftshi5* (SAIL_262_D04). Seeds were sterilized in 70% ethanol (with 0.05% Tween20) for 10 min and then washed twice in 95% ethanol and 100% ethanol. After the ethanol evaporated, the seeds were placed on 1/2 MS media (1/2 MS) agar plates supplemented with 50 µg ml^−1^ kanamycin. Plates were then cold-treated at 4°C for 48-h and allowed to germinate. Plants were grown at 22°C in a greenhouse with a light intensity of 300 µmol m^−2^s^−1^ under a 16-h-light/8-h-dark cycle. Measurement of leaf area was performed using standard protocols by LI-3000, A (LI-Cor Inc., Nebraska, USA).

### Isolation of *ftshi* mutants and segregation analysis

The T-DNA flanking sequence was amplified as described previously [35]. Heterozygous *ftshi4-1*, *ftshi4-2*, *ftshi1*, *ftshi2-1*, *ftshi2-2*, *ftshi2-3*, and *ftshi5* mutant plants were either self- or reciprocally crossed with wild-type. In both cases, the seeds produced were sown on soil, and plants were genotyped by PCR and phenotyped by analyzing seed development. The following primers were used for genotyping:

LB: 5′-GCTTCCTATTATATCTTCCCAAATTACCAATACA-3′ (for *ftshi1*, *ftshi2*, and *ftshi5*)

FtsHi1LP: 5′-GCCACAACCTCGTTGATTTCTTCT-3′

FtsHi1RP: 5′-CATACTGTTGGCTCAACATGGT-3′

FtsHi2-1RP: 5′-CCCTAACTCATCCCATTGTTCCCA-3′

FtsHi2-1LP: 5′-CCTCTGCATTTTCGTCACCT-3′

FtsHi2-2RP: 5′-CGTGCTGCTGAGTTGAAGAGA-3′

FtsHi2-2LP: 5′-GTACCTTCCTCAACCTCCTCATC-3′

FtsHi2-3RP: 5′-TCTTCTCTATCTCTGCTTCTC-3′

FtsHi2-3LP: 5′-CTGCTCCAACCATGCCATCTG-3′

FtsHi5LP: 5′-GGAAAGCTTTATGTTCCGGAG-3′

FtsHi2RP: 5′-TGCAGCAATAACTGCTGTCAC-3′

LB2: 5′-GATCGACCGGCATGCAAG-3′ (for *ftshi4-1*)

FtsHi4-1RP: 5′-CAATGTTCTACTCCAATCTGATGCC-3′

FtsHi4-1LP: 5′-TCTCTGTCTAGTTTCGTCCGTCG-3′

LB3: 5′-ATTTGCCGATTTCGGAAC-3′ (for *ftshi4-2*)

FtsHi4-2RP: 5′-GAGCAGAACTGCAAAACGTTC-3′

FtsHi4-2LP: 5′-CATTCCCGTCTGAAGAATCAG-3′

For phenotyping, siliques were dissected to count the ratio of white seeds to green seeds at 8 days after pollination (DAP). Plants producing 25% white seeds were scored as heterozygous and plants producing 100% green seeds were scored as wild type.

### Whole-mount preparations

Ovules of heterozygous *ftshi* mutants were cleared, as described by Liang *et al.* [13]. Briefly, siliques from the heterozygous *ftshi* mutants ranging from 2 to 12 DAP were dissected with hypodermic needles, and the ovules were immersed in HCG solution (80-g chloral hydrate, 10-ml glycerol, 30-ml H_2_O), mounted on microscopy slides after being cleared, and then observed under a Leica 5500 microscope equipped with DIC optics.

### TEM analysis of plastid development

Green (wild type) and white (*ftshi* mutants) ovules were isolated from the same heterozygous plant siliques when wild-type ovules developed to the torpedo stage. Ovules were punctured with a needle to ensure efficient fixative penetration. The ovules were fixed for 8 h in 200 mM phosphate buffer (pH 7.2) containing 4% glutaraldehyde at room temperature, followed by an overnight incubation in 2% OsO_4_. The samples were dehydrated in an ethanol series before being embedded in Spur. Ultrathin sections (100 nm) were stained for 15 min in uranyl acetate solution followed by 5 min in lead citrate. Ultrasections were observed under a transmission electron microscope (TEM) (model RILI H-7500) at 80 kV.

### Construction of the *FtsHi4* hairpin RNA expression vector

A 265-bp DNA fragment of *FtsHi4* gene was amplified by PCR with the following primer pairs: FF, 5′-CAGACGGGAATGACTGCT-3′ and FR, 5′-CGACAGGTCTCACGGGTT-3′. This fragment is upstream of the stop codon of the *FtsHi4* gene. The PCR products were cloned into the pENTR1A vector at the *Kpn*I and *Not*I sites, and then recombined into the destination vector pK7GWIWG2 with Gateway LR Clonase II Enzyme Mix (Invitrogen) to generate a hairpin RNA expression vector. This construct was introduced into *Arabidopsis* via *Agrobacterium*-mediated floral dipping [36].

### Genetic complementation

For the molecular complementation experiment, a 4941-bp genomic region containing the full-length *FtsHi4* gene and 866 bp upstream of the *FtsHi4* ATG start codon was amplified by PCR using the forward primer gFtsHi4F: 5′-GCTCTAGGTACCTTGTGTTGCTTTTTATGGGTCTC-3′ and the reverse primer gFtshi4R: 5′-GCTCTAGTCGACTTAAAAGAAATGGGTTGCCATCAAA-3′. The PCR product was cloned into the binary vector pCAMBIA1300 at the *Kpn*I and *Sal*I sites in the correct orientation. Constructs were verified by sequencing and used to transform *Arabidopsis* plants heterozygous for the mutant allele using floral dipping, as described previously [36]. The collected seeds were plated on 1/2 MS culture medium supplied with 25 mg l^−1^ hygromycin B, and the green seedlings were transplanted into soil.

### Real-time RT-PCR expression analysis

Total RNA was extracted according to the EASYspin Plant RNA kit manufacturer's instructions (Galen Biopharm, Beijing, China) with a DNase I (TaKaRa Bio, Ohtsu, Japan) treatment step. Total RNA (1 µg) was subjected to synthesis of single-stranded cDNA using the First Strand DNA Synthesis kit (Toyobo, Osaka, Japan). ACTIN primers were used to detect genomic DNA contamination. Relative quantification values for each target gene were calculated using the 2-⊿⊿CT method [37] using ACTIN as an internal reference gene to compare data from different PCR runs or cDNA samples (qRT-PCR). qRT-PCR analysis provided relative changes in gene expression, with the root normalized to a value of 1. Data were analyzed statistically using Student's *t*-test. The results shown are representative of two independent experiments, and within each experiment treatments were replicated three times unless otherwise stated. The primers used for the real-time RT-PCR analysis of *FtsHi4* gene expression were as follows: FtsHi4F, 5′-GGCTTACCGAGAAGCAGCTGTTG-3′; FtsHi4R, 5′-CTACTCTAGGAGCACATGCACGG-3′. The *Actin2* gene was used as a loading control. The primers were as follows: ACTIN2F, 5′-ATGTCTCTTACAATTTCCCG-3′. ACTIN2R, 5′-CCAACAGAGAGAAGATGACT-3′.

### Construction of the *FtsHi4*-GFP fusion and GFP transient assay

The N-terminal 300 bp of the *FtsHi4* coding region were amplified by PCR and fused in frame into the expression vector (pTH2) p35S-sGFP using *Sal*I and *Nco*I restriction endonucleases. The primers used were: sFtsHi4F (5′-GGACCTGGTCGACATGACGTTTTATATCTCGAGCTCG-3′) and sFtsHi4R (5′-GGACCTGCCATGGTAAGTCGTTCTCTTTCAGTTTCT-3′). We used *Arabidopsis* mesophyll protoplasts for the GFP transient expression assay. Briefly, well-expanded leaves from 3–4-week-old *Arabidopsis* plants were cut into 0.5–1-mm strips with fresh razor blades and digested in 5–10-ml cellulase/macerozyme solution (1–1.5% cellulase R10, 0.2–0.4% macerozyme R10 (Yakult Honsha, Tokyo, Japan), 0.4 M mannitol, 20 mM KCl, 20 mM MES, 10 mM CaCl_2_, and 0.1% bovine serum albumin (BSA) (Sigma A-6793; St. Louis, MO, USA), pH 5.7) for 3 h at 23°C. The enzyme solution containing protoplasts was filtered with a 35–75-µm nylon mesh and spun at 80×*g* for 2 min. The protoplasts were washed once in cold W5 solution (154 mM NaCl, 125 mM CaCl_2_, 5 mM KCl, 2 mM MES, pH 5.7), resuspended in cold W5 solution at 1–2×10^5^/ml, and maintained on ice for 30 min. The protoplasts were centrifuged at 80×*g* for 2 min and resuspended in MMg solution (0.4 M mannitol, 15 mM MgCl_2_, 4 mM MES, pH 5.7) at 1–2×10^5^/ml. A total of 10 µl of DNA (10–20 µg) aliquots and 100 µl of protoplasts were added to a 2-ml microfuge tube and mixed. PEG/Ca solution (110 µl of 40% PEG4000 (Fluka, #81240), 0.2 M mannitol, 1 mM CaCl_2_) was added, mixed, incubated at 23°C for 10 min, diluted with 0.44-ml W5 solution, and mixed gently. The mixture was centrifuged at 80×*g* for 1 min to remove the PEG, and the protoplasts were resuspended gently and diluted in 100 µl, followed by the addition of 1 ml of W5 to six-well plates. Protoplasts were then cultured for 6–16 h at 23°C. Cells exhibiting GFP signals were visualized using a confocal laser scanning microscope (LSM510; Carl Zeiss, Jena, Germany).

### Thylakoid membrane preparation

Thylakoid membranes were prepared as described previously [38]. Briefly, homogenized leaves in an ice-cold extraction buffer (400 mM sucrose, 50 mM HEPES-KOH, pH 7.8, 10 mM NaCl, and 2 mM MgCl_2_) were filtered through two layers of cheesecloth. The filtrate was centrifuged at 5000×*g* for 10 min. Following a wash with extraction buffer, the thylakoid pellets were re-centrifuged and finally suspended in extraction buffer. The chlorophyll contents were determined spectrophotometrically, as described by Porra *et al.* [39]. The isolated thylakoid membranes were used immediately.

### Antibodies and immunolocalization

The polyclonal antibody against FtsHi4 was raised in rabbits using the synthetic peptide sequence SETSGRVFARKSDY. Intracellular localization of FtsHi4 was determined as described previously [40]. *Arabidopsis* thylakoid membranes were suspended to a final concentration of 100 mg chlorophyll/ml. After 30-min incubation in 10 mM HEPES-KOH, pH 8.0, 10 mM MgCl_2_, 0.33 M sorbitol, and 1 mM PMSF, the thylakoid membranes were sonicated three times for 15 s on ice. After salt treatment (250 mM NaCl, 200 mM Na_2_CO_3_, 1 M CaCl_2_, or 6 M urea), the membranes were centrifuged at 100,000×*g* at 4°C for 2 h, washed twice with suspension buffer, and then subjected to immunoblot analyses.

### BN-PAGE, SDS-PAGE, and protein identification

BN-PAGE was performed according to Schägger *et al.* [41]. Following a wash with 0.33 M sorbitol and 50 mM Tris-HCl, pH 7.0, the thylakoid membranes were solubilized in 1% (w/v) DM (20% glycerol, 25 mM Tris-HCl, pH 7.0) at 0.5 mg chlorophyll/mL. After a 10-min incubation at 4°C and centrifugation at 12,000×*g* for 10 min, the supernatant was combined with 1/10 volume of 5% Serva blue G in 100 mM Tris-HCl, pH 7.0, 0.5 M 6-amino-η-caproic acid, and 30% (w/v) glycerol, and loaded on 6–12% acrylamide gradient gels with a thickness of 0.75 mm. For two-dimensional analysis, BN-PAGE lanes were excised and then soaked for 60 min in SDS sample buffer containing 5% β-mercaptoethanol and layered onto 15% SDS-PAGE gels containing 6 M urea with a thickness of 1 mm. After electrophoresis, proteins were exposed to antibodies, transferred to nitrocellulose membranes, and visualized using the enhanced chemiluminescence method [42].

### Measurements of chlorophyll contents

After being cleaned and weighed, the *Arabidopsis* plant cotyledons and leaves cultured for 3 weeks were ground into homogenate, along with a small amount of calcium carbonate, quartz sand and 1 ml of 95% ethanol. The extract was filtered to 10 ml in a brown volumetric flask with filter paper. The mortar, pestle and residue were washed several times with a small amount of 95% ethanol until no green was visible, after which ethanol was added to a final volume of 10 ml. The extract absorbance was measured with 95% ethanol as a control at 665 and 649 nm, respectively. Chlorophyll contents were calculated as follows based on the extract volume and material weight: Ca = 13.95A665–6.88A649, Cb = 24.96A649–7.32A665.

### Measurements of chlorophyll fluorescence

Fluorescence measurements were performed as described previously [43] using a portable fluorometer (PAM-2000, Walz, Fffeltrich, Germany). Before measurements, leaves were dark-adapted for 30 min. The minimum fluorescence yield (F_0_) was measured under measuring light (650 nm) with low intensity (0.8 µmol m^−2^ s^−1^). To estimate the maximum fluorescence yield (Fm), a saturating pulse of white light (3000 µmol m^−2^ s^−1^ for 1 s) was applied. The maximum photochemical efficiency of PSII was determined from the ratio of variable (*F_v_*) to maximum (*F_m_*) fluorescence (*F_v_/F_m_* = *(F_m_-F_o_)/F_m_*) [44]. All the above measurements were performed in a dark room under stable ambient conditions.

### Yeast two-hybrid assay

The coding sequences of *FtsHi1, FtsHi2, FtsHi4 and FtsHi5* were amplified using the gene-specific primers indicated below, and the PCR products were cloned into pGADT7 and pGBKT7 vector, respectively. The yeast two-hybrid assay was performed following the manufacturer's instruction (Clontech, California, USA).

FtsHi1F: GTCGGATCCATGGCGTCTATAGACAATGTTTTCTC

FtsHi1R: GTCCTCGAGTCATACTTGAGCATTGACGTC

FtsHi2F: GTCCATATGATGGCTTGTCGTTTCCCTCTG

FtsHi2R: GTCCCCGGGTTATGAACTGTTTCTTGCGGTAG

FtsHi4F: GTCCCCGGGATGACGTTTTATATCTCGAGCTC

FtsHi4R: GTCCCCGGGTTAAAAGAAATGGGTTGCCATC

FtsHi5F: CTGGAATTCATGATACTGCCCAATGTTTTGGAA

FtsHi5R: CTGGGATCCTTAGGTTGGTGCACTAAGAAGTGC

## Results

### Isolation and characterization of the *ftshi4* mutant

**Figure 1:**
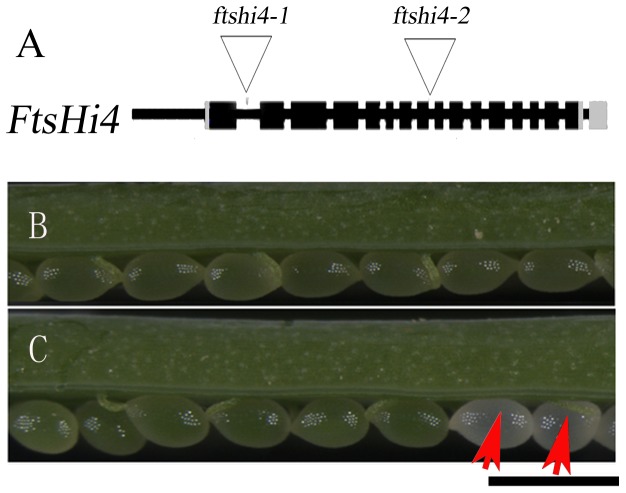
Isolation and characterization of *Arabidopsis ftshi4* mutant. A, Diagram of the T-DNA insertion position in the *FtsHi4* gene. Black boxes represent exons. The 5′ untranslated region (UTR) and 3′ UTR are shown in grey boxes. B, 9-DAP dissected wild-type silique. C, 9-DAP dissected *ftshi4* (+/−) mutant silique. Bar  = 1 mm.

To investigate chloroplast biosynthesis during embryogenesis in *Arabidopsis*, we generated an *Arabidopsis* T-DNA insertion population [35] and screened for mutant plants whose siliques contained roughly 25% albino embryos arrested at early embryogenesis; other seeds appeared green due to chlorophyll accumulation. As a result, a mutant plant that produced 24.3% albino seeds (117 of 481, 10 siliques) was identified (Fig. 1C; Table 1). When the mature seeds harvested from this plant were plated on 1/2 Murashige and Skoog (MS) medium containing 50 mg/L kanamycin (Km), the ratio of kanamycin-sensitive versus kanamycin-insensitive seeds was 1∶2 (231∶476, p>0.05), which is consistent with a single insertion line of embryo-lethal defects. We reciprocally crossed wild-type and mutant plants, and no white ovules were observed in the crossed siliques, suggesting that no parental effects were involved. Moreover, each of the Km-resistant plants produced approximately 25% white ovules, as observed in the mutant parents (n = 127). These results indicated that this mutation was recessive and monogenic, and that it affected embryo development but did not cause gametophytic defects.

**Table 1:** Segregation analysis of green and white seeds in developing siliques of heterozygous </emph>***ftshi*** **mutants and heterozygous** ***ftshi4*** **mutants containing** ***FtsHi4*** **genomic DNA.**

Parental genotype	Green Seeds	White seeds	Number examined	% of white seeds	χ^2^
*ftshi4*+/+	138	1	139	0.7	
*ftshi4-1*+/−	364	117	481	24.3	0.008
*tshi4-2*+/−	218	72	290	24.8	0
*gftshi4-1 gftshi4-8 gftshi4-12 gftshi4-22*	27 19 14 22	394 271 213 322	421 290 227 344	6.41 6.55 6.17 6.40	0.0005 0.008 0.007 0
*ftshi5*+/+	231	0	231	0	
*ftshi5*+/−	384	138	522	26.4	0.02
*ftshi2*+/−	180	2	182	1.1	
*ftshi2-1*+/−	496	150	646	23.2	1.00
*ftshi2-2*+/−	418	134	552	24.3	0.12
*ftshi2-2*+/−	344	116	460	25.2	0.001
*ftshi1*+/+	211	0	211	0	
*ftshi1*+/−	417	141	558	25.3	0.02

*gftshi4* indicates heterozygous *ftshi4* mutant containing *FtsHi4* genomic DNA.

To identify the T-DNA insertion site, we performed polymerase chain reaction (PCR)-based genomic walking in the transgenic line using *Hpa*I-digested genomic DNA as template (see Materials and Methods). One PCR product was obtained and sequenced, revealing the presence of a T-DNA insert 367 bp downstream of the ATG start codon of the At5g64580 gene locus. A BLAST search against NCBI indicated that this locus encodes an FstH-like protein named FtsHi4, which is composed of 17 exons and 16 introns, and that the T-DNA was inserted into the first intron (Fig. 1A). The T-DNA insertion position was also confirmed by amplification of the flanking region using primers specific to the T-DNA left border and *FtsHi4*. Thus, the mutant was designated *ftshi4-1*.

To further confirm the gene mutation responsible for the observed phenotypes, an independent T-DNA insertion mutant was obtained from the *Arabidopsis* Biological Resource Center (ABRC) (Salk_113657). The T-DNA was located in the eighth intron, as confirmed by PCR amplification and sequencing of the T-DNA flanking region (Fig. 1A). The Salk_113657 line showed the same phenotypes as *ftshi4-1*, which also produced 25% white ovules in the heterozygous line (Table 1). F_1_ plants from the cross between *ftshi4-1* and Salk_113657 produced 25% white seeds (Table 2), suggesting that these two mutants were allelic. Accordingly, Salk_113657 was named *ftshi4-2*.

**Table 2:** Analysis of reciprocal crosses between wild type and *ftshi* mutants.

Maternal	Paternal	Green Seeds	White Seeds	Number Examined	% of white seeds	χ^2^
*ftshi4*+/+	*ftshi4-1*+/−	203	1	204	0.49	
*ftshi4-1*+/−	*ftshi4*+/+	306	0	306	0	
*ftshi4*+/+	*ftshi4-2*+/−	225	0	225	0	
*ftshi4-2*+/−	*ftshi4*+/+	244	0	244	0	
*ftshi4-1*+/−	*ftshi4-2*+/−	325	107	432	24.8	0.003
*ftshi4-2*+/−	*ftshi4-1*+/−	230	73	303	24.1	0.089
*ftshi5*+/+	*ftshi5*+/−	172	0	172	0	
*ftshi5*+/−	*ftshi5*+/+	231	0	231	0	
*ftshi2*+/+	*ftshi2-1*+/−	193	1	194	0.52	
*ftshi2-1*+/−	*ftshi2*+/+	213	0	213	0	
*ftshi3*+/+	*ftshi3-2*+/−	160	0	160	0	
*ftshi2-2*+/−	*ftshi2*+/+	186	0	186	0	
*ftshi2*+/+	*ftshi2-3*+/−	176	1	177	0.56	
*ftshi2-3*+/−	*ftshi2*+/+	178	0	178	0	
*ftshi2-1*+/−	*ftshi2-2*+/−	318	102	420	24.3	0.079
*ftshi2-2*+/−	*ftshi2-1*+/−	202	68	270	25.2	0
*ftshi2-1*+/−	*ftshi2-3*+/−	431	140	571	24.5	0.047
*ftshi2-3*+/−	*ftshi2-1*+/−	263	85	348	24.4	0.034
*ftshi2-2*+/−	*ftshi2-3*+/−	142	49	191	25.7	0.016
*ftshi2-3*+/−	*ftshi2-2*+/−	243	76	319	23.8	0.176
*ftshi1*+/+	*ftshi1*+/−	240	1	241	0.4	
*ftshi1*+/−	*ftshi1*+/+	158	0	158	0	

To further verify whether the T-DNA insertion in At5g64580 was responsible for the seed abortion observed, a complementation vector containing a 4941-bp genomic DNA fragment spanning the *FtsHi4* gene was constructed and introduced into the heterozygous mutant plants by floral dipping. A total of 6.25% seed abortion was expected if a single copy transgene was introduced into the heterozygous plants and was located on a different chromosome than *FtsHi4*. Four independent hygromycin-resistant T_1_ transgenic plants were identified, all of which produced approximately 6.25% white seeds (Table 1). These results indicated that the introduced genomic DNA fragment completely rescued the mutant phenotype.

### *ftshi4* mutations lead to embryo arrest at the globular to heart-shaped transition stage

**Figure 2:**
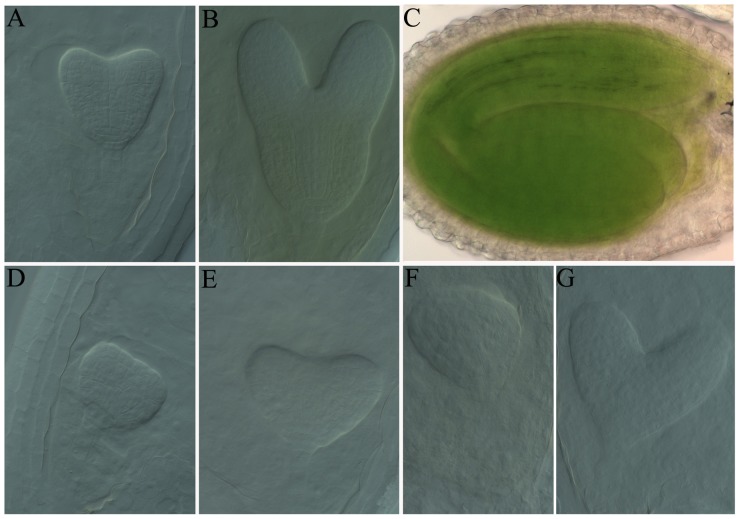
Development of *ftshi4* mutant embryos. A to C, Wild-type embryos from heterozygous *ftshi* plants undergoing normal development. A, Heart-shaped stage. B, Early torpedo stage. C, Mature embryo. D to G, Mutant embryos from heterozygous *ftshi4-1* plants were retarded and morphologically abnormal compared to wild-type embryos from the same silique. D, Mutant embryo development was retarded when wild-type embryo developed to the heart-shaped stage in the same silique. E, Mutant embryo showing abnormalities in the regions that develop an embryo axis and radicle compared with wild-type embryos that developed to the early torpedo stage in the same silique. F, Mutant globular embryo was morphologically abnormal when wild-type embryo reached maturity in the same silique. G, Mutant embryo was arrested at the heart-stage when wild-type embryo reached maturity in the same silique.

The observation that no homozygous *ftshi4* plants were recovered suggested that homozygous mutants are completely embryonic lethal. To investigate embryo development, we cleared the seeds from *ftshi4* heterozygous plants and observed them under a Nomarski microscope. At the globular embryo stage, which is the onset of chlorophyll accumulation [2], no obvious defects in embryo development were observed (n = 526). At the heart-shaped stage, the mutant embryos in the same siliques were mostly at the globular stage (Fig. 2A, D). When wild-type embryos reached the mature stage, about 80% (n≥60) of mutant embryos were arrested at the globular stage, whereas the remaining 20% were able to reach the heart-shaped stage, although an obvious abnormal division pattern was observed (Fig. 2C, F, G).

### Mutations in FtsHi4 affect plastid biogenesis and thylakoid formation

**Figure 3:**
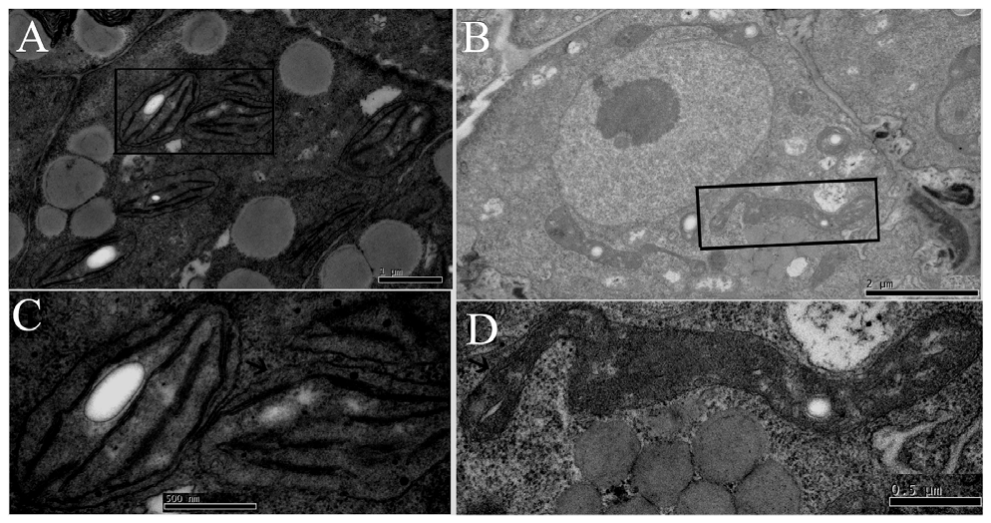
Transmission electron microscopy analyses of chloroplast biogenesis in the *ftshi4* mutant embryos. A, Wild-type torpedo embryo from a heterozygous *ftshi4-1* plant with well-developed chloroplasts showing thylakoid membranes beginning to stack into grana. B, Mutant embryo from the same heterozygous *ftshi4-1* silique with development-disrupted “plastids”. C, Enlargement of an above-described chloroplast indicated by an arrow.D, Enlargement of an above-described “plastid” indicated by an arrow.

Chloroplast differentiation occurs at the globular-to-heart transition stage during embryogenesis [2] when the embryos started to accumulate chlorophylls. Since the homozygous *ftshi4* mutants produced white ovules (Fig. 1C), we explored whether plastid development was impaired in the mutant during embryogenesis. We compared the plastid ultrastructure of the wild-type and the *ftshi4-1* mutant embryos in the same siliques of the heterozygous mutant plants at the torpedo stage, coincident with the stage when embryos turn green. In wild-type embryos, thylakoid membranes developed normally and began to stack into grana, an important indicator of plastids that have started to differentiate into spindle-shaped chloroplasts (Fig. 3A, F). In contrast, in *ftshi4-1* embryos, plastids were morphologically irregular, polymorphic, and poorly differentiated with very few internal membranes (Fig. 3B, G). No normally developed thylakoids were observed in mutant plastids. These observations indicated that plastid biogenesis and thylakoid differentiation were largely impaired in *ftshi4* mutant embryos.

### FtsHi4 is an integral thylakoid membrane protein

**Figure 4:**
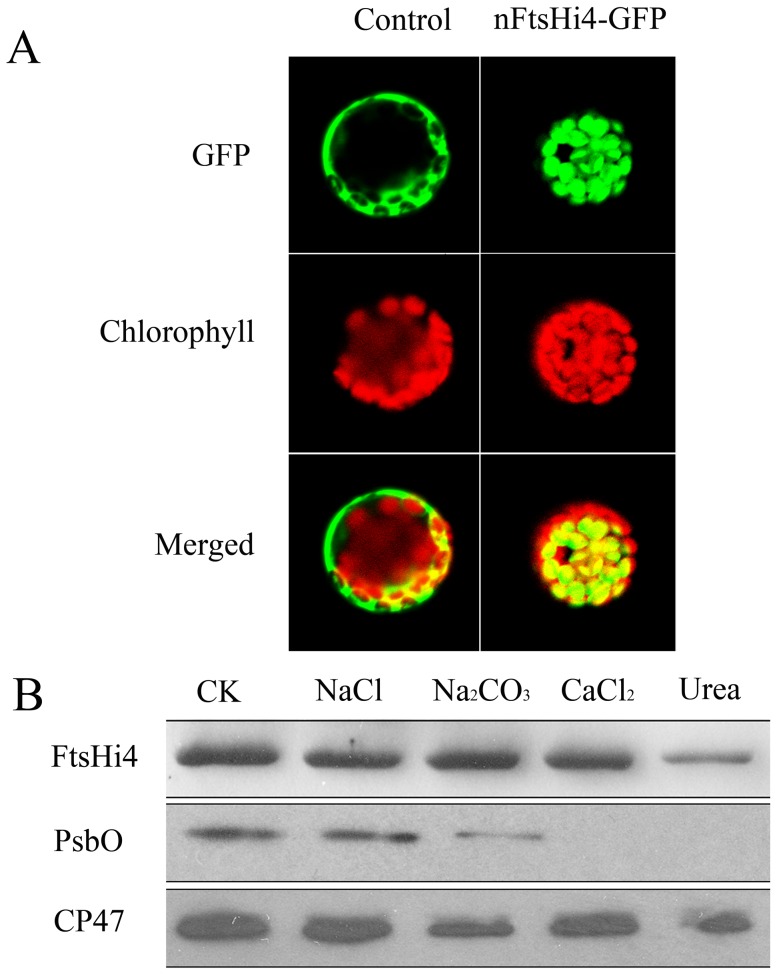
Subcellular and suborganellar localization of FtsHi4. A, *In vivo* targeting of green fluorescent protein (GFP) mediated by the FtsHi4 signal peptide in protoplasts. *Arabidopsis* protoplasts transformed by fusion between the FtsHi4 signal peptide and GFP. GFP fluorescence, chlorophyll autofluorescence, and merged images are shown. Free GFP was used as the control. B, Suborganellar localization of FtsHi4 protein. Thylakoid membranes were prepared from wild-type plants, fractionated by SDS-PAGE, transferred to polyvinylidene difluoride membranes, and visualized using antibodies raised against the FtsHi4 segment, PsbO (a 33-kDa luminal protein of PSII), or CP47 (a core protein of PSII). Membranes that were not subjected to salt treatment (CK) were used as controls.

Since plastid biogenesis was impaired in *ftshi4* mutants, as revealed by transmission electron microscopy (TEM), we speculated that FtsHi4 may be a chloroplast-localized protein. Analysis using the ChloroP 1.1 Server (http://www.cbs.dtu.dk/services/ChloroP/) software showed that FtsHi4 contained a targeting signal peptide for chloroplasts. To confirm localization, the N-terminal polypeptide of FtsHi4 containing the targeting signal peptide was fused to the N-terminus of green fluorescent protein (GFP) under control of the constitutive CaMV 35S promoter. The resulting construct was introduced into *Arabidopsis* leaf protoplasts. Green fluorescent signals were co-localized with the chlorophyll autofluorescence in the transformed protoplasts, whereas control GFP lacking the FtsHi4 signal peptide was retained in the cytosol (Fig. 4A). These results indicated that FtsHi4 was targeted to chloroplasts.

To further investigate sub-organelle localization of the FtsHi4 protein, the thylakoid membrane fractions were isolated from wild-type plants and treated with alkaline and chaotropic salts to release membrane-associated proteins [40]. In this assay, PsbO (a 33-kDa luminal protein of PSII) and CP47 (a core protein of PSII) were used as markers to distinguish peripheral and integral membrane proteins, respectively. We found that FtsHi4 was retained in the membrane fraction, behaving similar to the integral protein CP47 (Fig. 4B). These results indicated that FtsHi4 was localized to the thylakoid as an integral membrane protein, even though it contains no predicted transmembrane domains.

### PSII protein accumulation is defective in the *ftshi4* mutant

**Figure 5:**
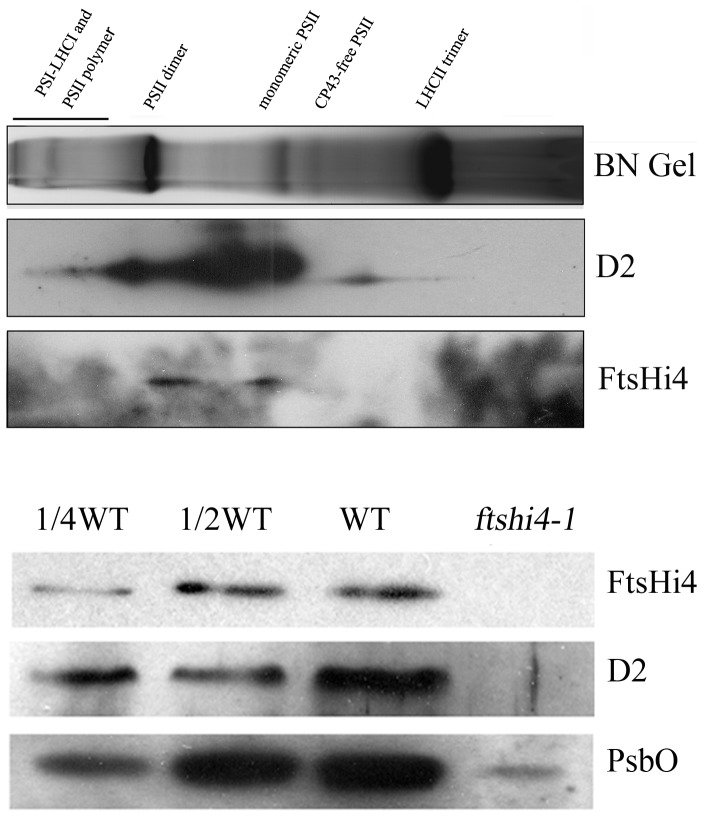
D2 and PsbO protein accumulation is defective in the *ftshi4* mutant. A, Detection of the PSII core subunit D2 and FtsHi4 by immunoblotting on two-dimensional gel electrophoresis. Membrane samples were solubilized with DM and separated in the first dimension on a blue native gel followed by SDS-PAGE in the second dimension. D2 and FtsHi4 proteins were detected using appropriate antibodies. The separated complexes are designated as: I, PSII supercomplexes; II, monomeric PSI and dimeric PSII; III, monomeric PSII; IV, dimeric cytochrome b6/f dimmer; V, trimeric LHCII; and VI, monomeric LHCII. B, Immunoblot analyses for the accumulation of D2, PsbO and FtsHi4 PSII proteins in wild-type and mutant ovules from heterozygous *ftshi4-1* mutant plants. The thylakoid membrane proteins were fractionated by SDS-urea-PAGE, and the blots were probed using antibodies raised against D2, PsbO, or FtsHi4.

Chloroplast thylakoid membranes contain a large number of proteins and protein complexes, such as photosystem I (PSI) and PSII, which play various roles in photosynthesis. FtsH proteins were previously shown to be localized near PSII at grana, and are responsible for turnover of the PSII D1 protein [28], [45]. Additionally, FtsHi4 shows a high degree of similarity to FtsH proteins [33]. Thus, we investigated whether FtsHi4 was also localized to PSII-enriched thylakoid membranes. To accomplish this, the thylakoid membrane protein complexes isolated from 4-week-old leaves were separated on blue native (BN)-PAGE gel, and the separated protein complexes corresponding to PSII supercomplexes (band I), monomeric PSI and dimeric PSII (band II), monomeric PSII (band III), dimeric cytochrome b6/f dimer (band IV), trimeric LHCII (band V), and monomeric LHCII (band VI) were fractionated by SDS-PAGE followed by immunoblotting using anti-FtsHi4 and -D2 antibodies. D2 protein is reported to assemble into the PSII complex [46]. Figure 5 showed that the D2 protein was mainly found at band II and III, and the FtsHi4 protein migrated in two protein complexes of ∼460 and ∼240 kDa, respectively, which also corresponded to the PSII dimer and monomer (Fig. 5A). Although some of the D2 complexes migrated at sizes similar to FtsHi4, the overall pattern differed between complexes. These results suggested that FtsHi4 forms a complex.

To increase our understanding of how defects in thylakoid membrane biogenesis caused by the loss-of-function of FtsHi4 affected PSII protein accumulation in the *ftshi4* mutant, immunoblot analyses were conducted using the thylakoid proteins extracted from green and white ovules isolated from the heterozygous *ftshi4-1* mutant plants. Accumulation of the FtsHi4 protein was non-detectable in the white ovules, and the two PSII proteins D2 and PsbO were completely lost or decreased significantly, respectively (Fig. 5B). These results indicated that the *ftshi4* mutation caused a defect in PSII protein complex formation.

### Knock-down of *FtsHi4*

**Figure 6:**
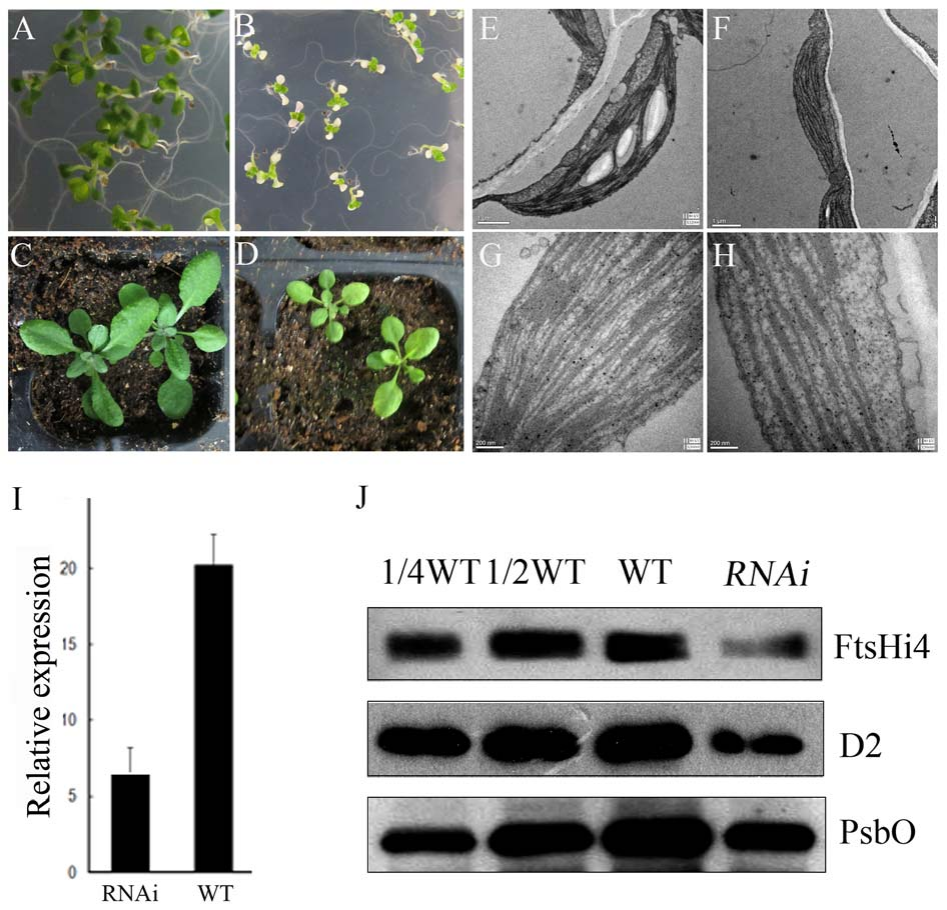
Down regulation of FtsHi4 by RNAi leads to defects in chloroplast development. A, 2-week-old seedlings of WT grown on half MS medium. B, 2-week-old seedlings of RNAi-*FtsHi4* line grown on half MS medium. C, 5-week-old plants of WT grown in soil. D, 5-week-old plants of RNAi-*FtsHi4* line grown in soil. E, Chloroplasts from a 5-week-old leaf of wild-type plant. These chloroplasts are well developed, with redundant grana interconnected by stroma thylakoids. Enlargement of such a chloroplast is shown in (G). F, Plastids from 5-week-old leaf of RNAi-*FtsHi4* plants, these plastids had straight thylakoids but lacked granal lamellae. Enlargement of such a plastid is shown in (H). ItoJ, qRT-PCR and immunoblot analysis showing expression levels of FtsHi4 in leaves excised from 2-week seedlings described in A and B. Chloroplast proteins were further detected in 2-week seedlings of the RNAi mutant using a specific antibody.

To further confirm the function of *FtsHi4*, the gene was silenced by double-stranded RNAi using a 265-bp cDNA fragment with 100% specificity to *FtsHi4*. The expression level of *FtsHi4* was examined in transgenic plants using qRT-PCR, which revealed a significant down-regulation of the *FtsHi4* gene in the RNAi line compared with WT plants (Fig. 6I). Further immunoblot analysis using an anti-FtsHi4 antibody confirmed the lower signal intensity in total protein preparations (Fig. 6J). Interestingly, the RNAi-*FtsHi4* mutant showed a strong phenotype in leaf color. Unlike the wild type, which had green cotyledons and mature leaves, the mutant plants had white cotyledons and yellowish leaves (Fig. 6A, B). In addition, the mutant plants were smaller than WT plants throughout their life cycle (Fig. 6C, D). Under normal conditions, growth was significantly reduced in RNAi plants. As shown in Figure S1, the leaf area of the RNAi plants was ∼40% smaller than WT plants 26 d after germination.

Ultrastructural changes in the chloroplasts of the RNAi-*FtsHi4* plants were also assessed. The 5-week-old leaves from wild type and the RNAi-*FtsHi4* mutant were sectioned to examine their chloroplast structure. The mutant plastids had straight thylakoids but lacked granal lamellae (Fig. 6E–H). We also analyzed thylakoid membrane protein accumulation of D2 and PsbO with specific antibodies, and found that protein levels of the RNAi-*FtsHi4* mutant decreased significantly compared with wild type (Fig. 6J).

### Defects in PSII functioning in RNAi mutant plants

**Figure 7:**
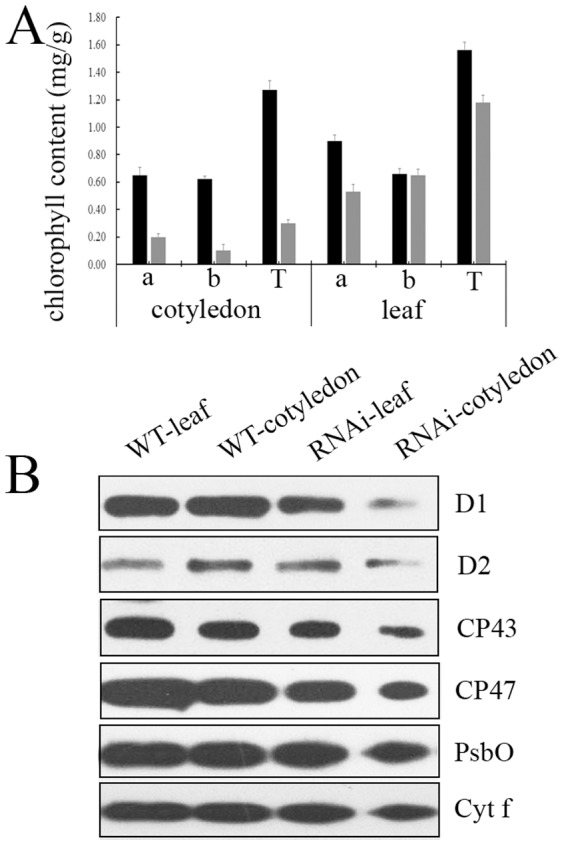
Defects in the PSII complex of the RNAi-*Ftshi4* mutant block energy transfer within PSII. A, The chlorophyll concentrations of wild-type and RNAi-*FtsHi4* mutant cotyledons and true leaves. B, Immunoblot analyses for the accumulation of D1, D2, CP43, CP47, PsbO, and Cyt f proteins in wild-type and RNAi-*FtsHi4* mutant cotyledons and true leaves. The thylakoid membrane proteins were fractionated by SDS-urea-PAGE, and the blots were probed using antibodies raised against D1, D2, CP43, CP47, PsbO, or Cyt f, respectively.

Since transgenic RNAi mutant plants displayed chlorosis in both cotyledons and leaves, we measured the chlorophyll contents in RNAi mutant plants. The results showed that both chlorophyll a and chlorophyll b were significantly reduced in the cotyledons of *FtsHi4*-RNAi mutant (0.20 mg/g in RNAi plants vs. 0.68 mg/g in WT for chlorophyll a; 0.1 mg/g in RNAi plants vs. 0.65 mg/g in WT for chlorophyll b). Unlike cotyledons, chlorophyll a levels in the RNAi plant leaves decreased to 64% compared to WT (0.58 vs. 0.9 mg/g), but the chlorophyll b content was similar in these two genotypes. Consequently, total chlorophyll was significantly reduced in the RNAi plants (Fig. 7A).

Fv/Fm (the ratio between variable fluorescence and maximum fluorescence) reflects the maximum potential capacity of the photochemical reactions of PSII [47]. Analyses of the chlorophyll fluorescence Fv/Fm ratio with dark-adapted leaves revealed a significant reduction in RNAi plants compared with WT (0.68 vs. 0.85), suggesting that FtsHi4 knock-down affected energy transfer within PSII.

On the other hand, we analyzed the accumulation of proteins essential for photosynthesis with corresponding antibodies in the transgenic RNAi plants (Fig. 7B). The results demonstrated that levels of D1, D2, CP43 and CP47, the PSII core subunits, Cyt f (the subunit of cytochrome b6f complex) and PsbO (the subunit of the oxygen-evolving complex) were significantly reduced in cotyledons of the RNAi plants compared with WT. Additionally, D1, CP43, and CP47 showed a reduction in leaves, but to a lesser degree than in cotyledons. No significant changes were found for D2, PsbO and Cyt f in leaves (Fig. 7B)

### *FtsHi4* gene expression pattern

**Figure 8:**
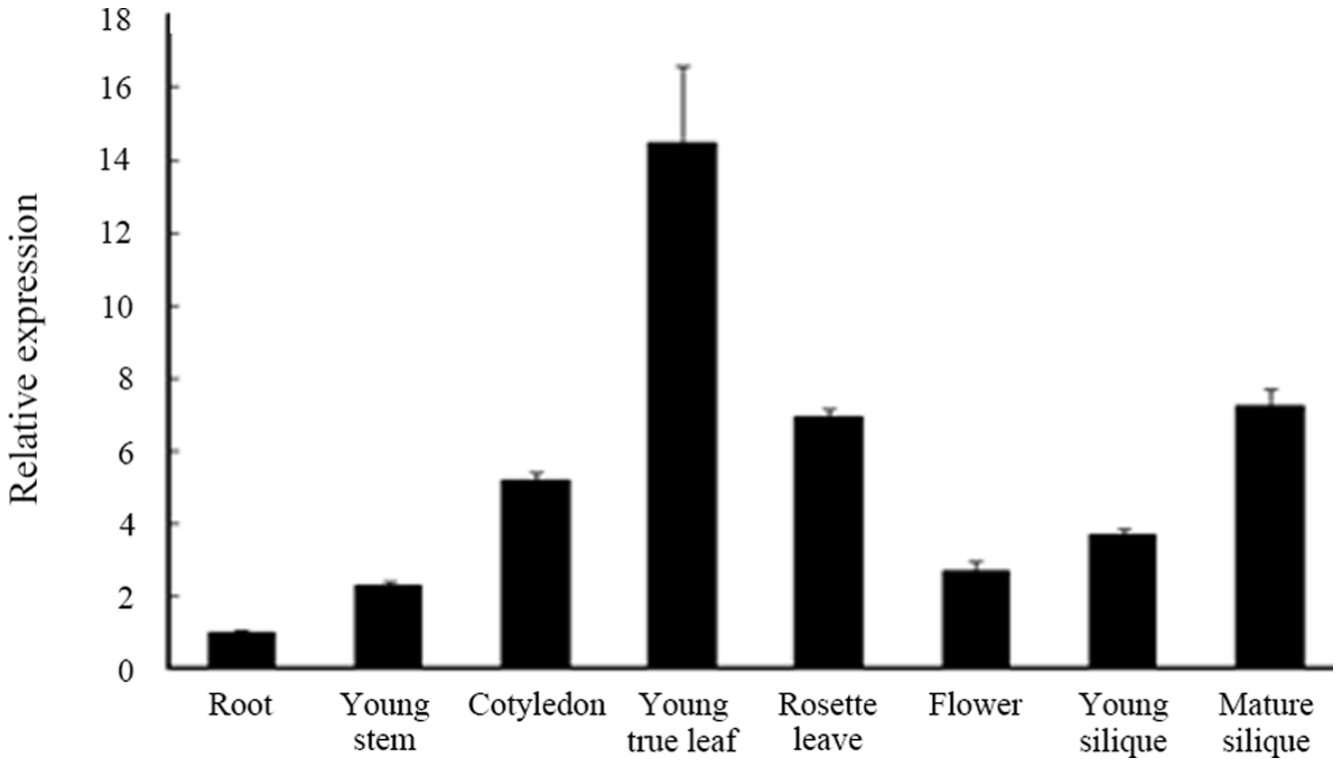
Expression patterns of *FtsHi4* gene in *Arabidopsis*. Total RNA was extracted from different tissues of 40-day-old wild-type plants grown in soil. Gene-specific primers were used to detect *FtsHi4* transcripts. The *ACTIN2* gene was used as a control.

Expression analyses of the *FtsHi4* gene in *Arabidopsis* using qRT-PCR with gene-specific primers showed that *FtsHi4* transcripts were ubiquitously present in organs, including roots, stems, cotyledons, young true leaves, rosette leaves, flowers, young siliques and mature siliques (Fig. 8). *FtsHi4* transcripts were most abundant in young leaves and present at the lowest levels in roots.

### *ftshi1*, *ftshi2* and *ftshi5* mutants resemble *ftshi4* phenotypes

Since all five FtsHi proteins represent a subclade, we generated T-DNA insertion mutants for the other four genes from ABRC (SAIL_262_D04 for *FtsHi1*, named *ftshi1*; *emb2083-1*, *emb2083-2*, and *emb2083-4* for *FtsHi2*, named *ftshi2-1*, *ftshi2-2*, and *ftshi2-3*, respectively; GK_723C06 and GK_555D09 for *FtsHi3*, named *ftshi3-1* and *ftshi3-2*, respectively; and *emb2458* for *FtsHi5*, named *ftshi5*and) (Fig. S2A). After genotyping by PCR using a combination of gene- and T-DNA left border-specific primers, no homozygous mutants were identified from these lines, suggesting that homozygous mutants may be embryo-lethal. The T-DNA insertion lines for FtsHi3 did not show any visible phenotypes under our conditions (data not shown). Similar to *ftshi4*, approximately 25% of the ovules in those heterozygous lines of *ftshi1*, *ftshi2*, and *ftshi5* were albinos with an embryo-lethal phenotype (Fig. S2C, D, E; Table 1). Reciprocal crosses between wild-type and heterozygous mutant plants showed that all of these mutations were recessive without maternal or paternal effects (Table 2).

To investigate embryo development of these mutants, ovules from heterozygous mutant plants were cleared in HCG solution and observed under a differential interference contrast (DIC) microscope. Similar to *ftshi4*, mutants of *ftshi1*, *ftshi2*, and *ftshi*5 exhibited no evident defects prior to the globular stage, whereas abnormal development was observed when wild-type embryos developed to the heart-shaped stage (Fig. S3). At the mature stage, ∼80% (n≥60) of mutant embryos were arrested at the globular stage, and the remaining 20% reached the heart-shaped stage with an abnormal division pattern (Fig. S3C, D, G, H, K, L).

Since these mutants showed embryo-developmental defects similar to *ftshi4*, we also examined plastid morphogenesis of the mutants during embryo development. Similar to *ftshi4-1*, these mutant plastids were morphologically polymorphic and accumulated very few internal membranes (Fig. S4). These observations indicated that FtsHi1, FtsHi2, FtsHi4, and FtsHi5 were required for plastid development during embryogenesis.

### FtsHi4 physically interacts with FtsHi2 in yeast cells

In Arabidopsis, two types of FtsH isomers, type A (FtsH5/FtsH1) and type B (FtsH2/FtsH8), form the FtsH heterocomplexes [24], [31], [55]. In this study, each of the *ftshi* mutants showed very similar phenotype (Fig. 2; Fig.S3) and two protein complexes with different molecular weight were observed in the BN gel analysis when anti-FtsHi4 antibody was used for immunobloting (Fig. 5). Therefore, it's interested to investigate whether the four FtsHis proteins, including FtsHi1, FtsHi2, FtsHi4 and FtsHi5, could interact each other using yeast two-hybrid system. The results showed that there was an interaction between FtsHi2 and FtsHi4. Interestingly, each of these two proteins interacted with itself, respectively (Fig. S5; Table S1).

## Discussion

In this report, we described the biological function of *FtsHi4*, an *Arabidopsis FtsH*-like gene involved in embryo development by affecting thylakoid biogenesis, using a T-DNA insertion *Arabidopsis* mutant. FtsHi4 encodes a thylakoid membrane-associated protein, the disruption of which caused a failure in thylakoid formation and led to an embryo-lethal phenotype.

FtsHi4 displays a high degree of similarity to FtsH proteins, a type of Zn^2+^-metalloprotease that degrades short-lived proteins and misassembled membrane proteins, thus contributing to cellular regulation at the level of protein stability and membrane protein quality control [48]. *ftsH* was first isolated from *E. coli* as a temperature-sensitive and cell-division-defective mutant [49]. *E. coli* FtsH is a membrane-bound protein containing two transmembrane segments at the N-terminus and a cytoplasmic region that includes an ATPase and a protease domain [50]. In *E. coli*, FtsH functions as both a protease and a chaperone. The lack of a Zn-binding motif in FtsHi suggests that these proteins function as chaperones rather than having protease activity [32].

Among the 12 *Arabidopsis* FtsHs, 7 have been characterized functionally. FtsH6 contributes to the degradation of Lhcb3 during dark-induced senescence and Lhcb1 and Lhcb3 during high-light acclimation [51]. FtsH1, FtsH5, FtsH2, and FtsH8 form the FtsH complex, which is involved in the repair of PSII (particularly turnover of the D1 protein) [25], [29], [31]. Single FtsH protein mutants are variegated, but not embryo lethal, although the double mutants of *atftsh2/atftsh8* and *atftsh1/atftsh5* are embryo lethal or seedling lethal, respectively [31]. For example, the *ftsh2* mutant and *ftsh*5 mutant display a variegation phenotype and are sensitive to photoinhibition [52]–[54]. However, in the current study, the *ftshi4* mutant was arrested at the globular to heart-shaped transition (Fig. 2D–G). Interestingly, mutants of other FtsHi proteins, including Ftshi1, Ftshi2, and Ftshi5, showed similar phenotypes during embryo development [34] (Figs. 2, 3). The *FtsH1, FtsH5, FtsH2*, and *FtsH8* genes are expressed ubiquitously with similar expression profiles, particularly in green organs; however, expression is low in the roots [24], [55]. At the protein level, FtsH2 is the most abundant FtsH protein in chloroplasts, followed by FtsH5, FtsH8, and FtsH1 [56]. *FtsHi4* was expressed in almost all organs examined (Fig. 8). These observations suggest that in contrast to *FtsH* genes, the expression of *FtsHi4* is not confined to green organs. Both the phenotypic difference in embryo development between the *ftsh* and *ftshi* mutants and the difference in expression profiling between *FtsH* and *FtsHi* genes suggests that these two proteins play different biological roles during chloroplast development. Although functional redundancy between FtsH proteins has been reported, there was no apparent functional redundancy between FtsHi proteins in this study. This is based on the observation that each *ftshi* mutant showed similar embryo lethality, suggesting that FtsHi forms a homo- or hetero-complex. In agreement with this, we found that FtsHi4 formed two protein complexes of ∼460 and ∼230 kDa, respectively, and the migration pattern of the FtsHi4 complexes differed markedly from that of D2, although some of the D2 complexes migrated to positions similar to the FtsHi4 complexes (Fig. 5). Moreover, it was also found that FtsHi2 and FtsHi4 interacted each other and each of these two proteins interacted with itself in yeast cells (Fig. S5). These results suggest that the FtsHi4 protein is not co-localized with D2, but functions as a complex. However, this requires further investigation using protein-protein interaction experiments.

Characterizing the biochemical function of FtsHi proteins will increase our understanding of how these proteins regulate plastid development. Unfortunately, we did not examine both prokaryotic and eukaryotic expression of FtsHi4 polypeptides in *E. coli* cells and yeast cells, respectively. Thus, we could not biochemically investigate whether FtsHi4 acts as a molecular chaperone or protease during thylakoid membrane biogenesis. However, there was a complete loss in D2 and a significant reduction in PsbO protein accumulation in the *ftshi4* mutant embryos (Fig. 5B). TEM analyses revealed the absence of normally developed thylakoids in mutant embryos (Fig. 3B, G). Thylakoid biogenesis is a complicated process involving physical aspects of formation and differentiation, genetic transmission, signaling cascades, and protein import and sorting. For example, mutations in genes controlling the upstream stages of thylakoid protein assembly cause the loss of thylakoid membrane proteins. Such mutants include *csr1* (defective in the targeting pathway to the thylakoids), *hcf7* and *cps* (defective in RNA metabolism), and *cpSecY* (defective in chloroplast translation) [57]–[59]. It has also been suggested that the nuclear- and plastid-encoded proteins must be targeted to the correct position on the thylakoid with the help of thylakoid-targeting machinery to ensure correct assembly of the thylakoid protein complexes [60]. The phenotypes of these mutants were characterized by abnormal plastid biogenesis and, in most cases, early seedling lethality. The formation of bilayer structures in the thylakoid membrane is an energetically demanding process that depends on the presence of LHC or other membrane proteins, which must be delivered to and combined with the lipid phase [61]. Thus, they likely lack some membrane proteins (e.g., PSII proteins), which may lead to failed thylakoid membrane biogenesis.

The proteins responsible for thylakoid protein complex formation in *Arabidopsis* play important roles in plastid differentiation. Examples of such proteins include HCF136, which is essential for the stability of PSII in *Arabidopsis* [62], ALB3, a subunit of the thylakoid Sec protein transport system in *Arabidopsis* [63], [64], APG2, a component of the *Arabidopsis* ΔpH-dependent thylakoid protein transport machinery [65], and TerC, a bacterial homologue in *Arabidopsis* involved in tellurite resistance [60]. Therefore, it is possible that the failed thylakoid membrane biogenesis in the *ftshi4* mutant embryos may have been directly caused (at least in part) by an impaired PSII protein complex, which ultimately caused embryo developmental arrest.

The role of plastids during embryogenesis remains unclear, but likely involves the synthesis of metabolites to nourish embryos or the production of signals to regulate nuclear genes. To date, a number of *Arabidopsis* mutants defective in plastid development have been reported. These mutants are grouped into four classes, one of which includes mutants that are truly embryo-lethal, with embryo development arrested at the globular-to-heart transition stage [23], such as the *emb1211* mutant, which is affected in early plastid differentiation [13], the protein import machinery *tic110* mutant [7], and the lysophosphatidic acid acyltransferase 1 mutant [22], [66]. Based on analysis of the biological processes of these genes, only those involved in basal cellular functions, such as chloroplast translation machinery and membrane biogenesis, are required for normal embryo development [13]. In this study, we found that mutations in *FtsHi* proteins (excluding *FtsHi3*) caused embryo arrest at the transition from the globular to heart-shaped stage, and that these mutants were defective in chloroplast biogenesis and thylakoid formation during embryogenesis (Figs. 2 and 3). These observations indicate that normal functioning of FtsHi proteins is essential for the formation of functional chloroplasts, and these four FtsHi proteins are indispensable for chloroplast biogenesis.

To further explore the possible functions of FtsHi proteins, we examined FtsHi proteins in the ATTEDII co-expression database and found that they are highly associated with each other, but not with other FtsH proteins. Interestingly, some proteins (such as pTAC12/HEMERA) are co-expressed with FtsHi proteins. It was reported that TAC12, together with other TAC members, plays a role in the phytochrome-dependent light signaling pathway to regulate PEP activity and plastid gene expression [67], [68]. In addition, searching of the PPDB proteome database showed that FtsHi1 and FtsHi2 are found in plastids. This result suggests that the FtsHi proteins may be involved in the regulation of plastid gene expression, thereby affecting chloroplast development.

In summary, the impaired accumulation of PSII proteins in the *ftshi4* mutant may be caused by failed functional thylakoid membrane formation. Consequently, these defects may have blocked plastid differentiation into functional chloroplasts at the globular to heart-shaped transition stage. However, the role of FtsHi4 and other FtsHi proteins in chloroplast biogenesis in *Arabidopsis* requires further investigation.

## Supporting Information

Figure S1 **Growth kinetics of the RNAi-** ***Ftshi4*** **mutant plants.** Values are averages ±S.E. of at least six replicated experiments.
(TIF)

Figure S2 **Isolation and characterization of** ***Arabidopsis ftshi1, ftshi2, and ftshi5*** **mutants.** A, Diagram of the T-DNA insertion position in the *FtsHi1, FtsHi2, and FtsHi5* genes. Black boxes represent exons. The 5′ untranslated region (UTR) and 3′ UTR are shown in grey boxes. B, 9-DAP dissected wild-type silique. C, 9-DAP dissected *ftshi1* (+/−) mutant silique. D, 9-DAP dissected *ftshi2* (+/−) mutant silique. E, 9-DAP dissected *ftshi5* (+/−) mutant silique. Arrows indicate white ovules with no chlorophyll accumulation. Bar  = 1 mm.
(TIF)

Figure S3 **Embryo development of** ***ftshi1*** **,** ***ftshi2*** **, and** ***ftshi5*** **mutants.** Mutant embryos from heterozygous *ftshi1* plants (A–D), heterozygous *ftshi2* plants (E–H), and heterozygous *ftshi5* plants (I–L) were retarded and morphologically abnormal compared to wild-type embryos from the same silique. A, E, and I, Mutant embryo development was retarded when wild-type embryo developed to the heart-shaped stage in the same silique. B, F, and J, Mutant embryos showed abnormalities in the regions that developed an embryo axis and radicle compared to wild-type embryos that developed to the early torpedo stage in the same silique. C, G, and K, Mutant globular embryos were morphologically abnormal when wild-type embryos reached maturity in the same silique. D, H, and L, Mutant embryos were arrested at the heart-stage when wild-type embryos reached maturity in the same silique. The wild-type embryo development is the same as in Figure 2.
(TIF)

Figure S4 **Transmission electron microscopic analysis of plastid development of** ***ftshi1*** **,** ***ftshi2*** **, and** ***ftshi5*** **mutants.** A, B, and C, Mutant *ftshi1*, *ftshi2-1*, or *ftshi5* embryos with development-disrupted “plastids”. Enlargements of the above-described “plastids” are shown in D, E, or F, respectively, and indicated by arrows. Wild-type embryos from each of the same heterozygous *ftshi1*, *ftshi2-1*, and *ftshi5* siliques are the same as in Figure 3 and therefore not shown.
(TIF)

Figure S5 **Yeast two-hybrid assay of FtsHi2 and FtsHi4 protein.** Yeast cells transformed with the corresponding vectors grew on the -Trp/-Leu medium (A) and -Ade/-His/-Leu/-Trp medium (B). Numbers of 1-10 represent the yeast hybrid with FtsHi proteins as baits or preys, respectively, which is described as following. 1, bait pGBKT7-53 and prey pGADT7-T. 2, bait pGBKT7-FtsHi2 and prey pGADT7-FtsHi2. 3, bait pGBKT7-FtsHi4 and prey pGADT7-FtsHi4. 4, bait pGBKT7-FtsHi2 and prey pGADT7-FtsHi4. 5, bait pGBKT7-FtsHi4 and prey pGADT7-FtsHi2. 6, bait pGBKT7-FtsHi2 and prey pGADT7. 7, bait pGBKT7-FtsHi4 and prey pGADT7. 8, bait pGBKT7 and prey pGBKT7-FtsHi2. 9, bait pGBKT7 and prey pGBKT7-FtsHi4.10, bait pGBKT7-Lam and prey pGADT7-T.
(TIF)

Table S1 **The interactions between FtsHi proteins in yeast cells.**
(DOC)
