# Correction: FtsHi4 Is Essential for Embryogenesis Due to Its Influence on Chloroplast Development in *Arabidopsis*

**DOI:** 10.1371/journal.pone.0229232

**Published:** 2020-02-12

**Authors:** Xiaoduo Lu, Dongyuan Zhang, Shipeng Li, Yanping Su, Qiuju Liang, Hongyan Meng, Songdong Shen, Yunliu Fan, Chunming Liu, Chunyi Zhang

In Fig 7B of this article [1], the PsbO data were duplicated in error as representing the Cyt f results. In the updated figure provided here, the Cyt f panel has been replaced with the correct image from the original experiment. The original uncropped blot images supporting Fig 7B are provided in Supporting Information S1–S6 Files of this notice. A member of *PLOS ONE*’s Editorial Board confirmed that the updated figure supports the results as reported in the original article.

**Fig 7:**
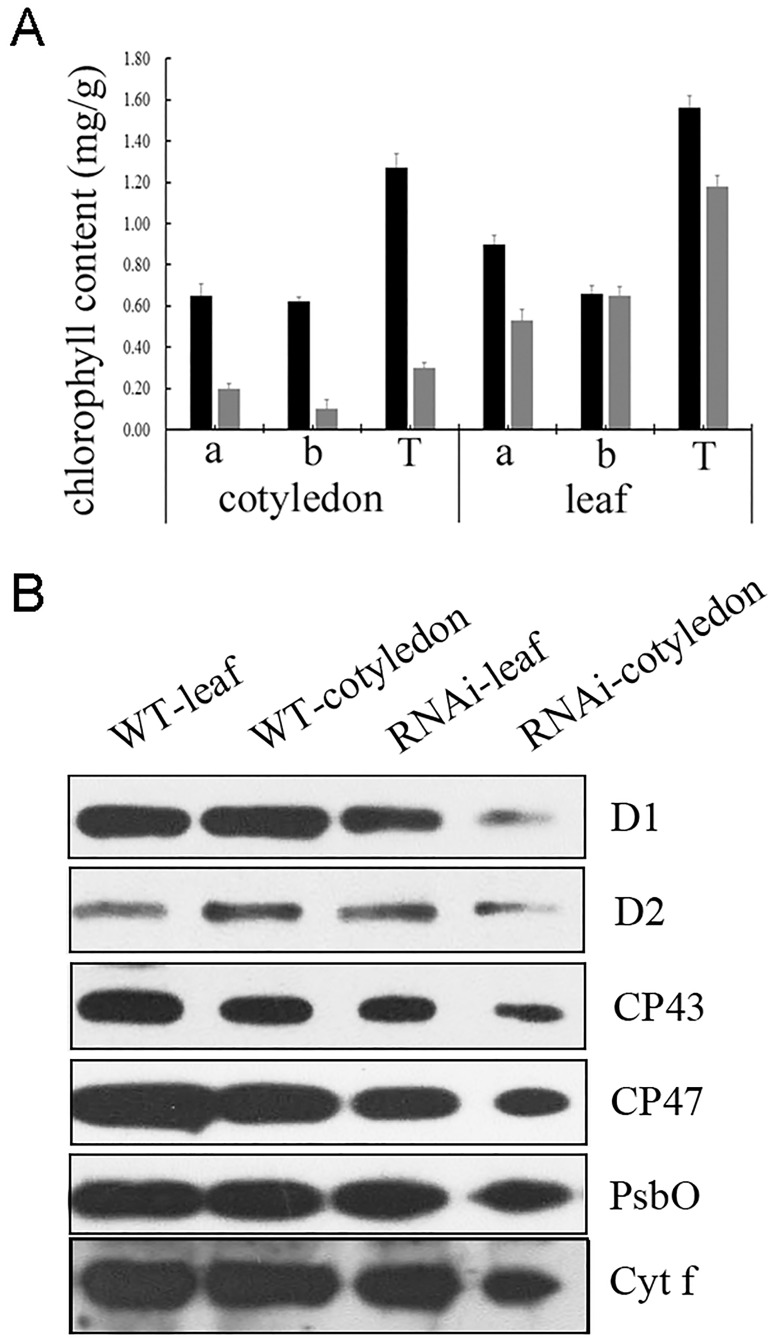
Defects in the PSII complex of the RNAi-*Ftshi4* mutant block energy transfer within PSII. A, The chlorophyll concentrations of wild-type and RNAi-*FtsHi4* mutant cotyledons and true leaves. B, Immunoblot analyses for the accumulation of D1, D2, CP43, CP47, PsbO, and Cyt f proteins in wild-type and RNAi-*FtsHi4* mutant cotyledons and true leaves. The thylakoid membrane proteins were fractionated by SDS-urea-PAGE, and the blots were probed using antibodies raised against D1, D2, CP43, CP47, PsbO, or Cyt f, respectively.

In addition, details as to the antibodies used for western blot experiments were not reported in the Material and Methods section. The antibodies used to detect the D1, D2, CP43, PsbO and Cyt f proteins were kindly provided by Dr. Lixin Zhang (College of Life Science, Henan University), and the details of those antibodies were described in a previous study [2]. The polyclonal antibody against FtsHi4 was raised in rabbits using the synthetic oligopeptide of SETSGRVFARKSDY, a part of the FtsHi4 protein. Specificity of the antibody was verified by the absence of FtsHi4 protein expression in the *ftshi4* mutant (Figure 5 of [1]).

Except for the image data provided here in support of Fig 7B, the raw data underlying results reported in this article [1] are no longer available.

The authors apologize for the error in the published article.

As of the date of this notice, the corresponding author (Chunyi Zhang) is affiliated with the Institute of Crop Science, Chinese Academy of Agricultural Sciences, Beijing 100081, P. R. China.

## Supporting information

S1 File Original image for the D1 western blot in Fig 7B. (TIF)

S2 File Original image for the D2 western blot in Fig 7B. (TIF)

S3 File Original image for the Cp43 western blot in Fig 7B. (TIF)

S4 File Original image for the Cp47 western blot in Fig 7B. (TIF)

S5 File Original image for the PsbO western blot in Fig 7B. (TIF)

S6 File Original image for the Cyt f western blot in Fig 7B. (TIF)
