# Supplementary figures and images for: FtsHi4 Is Essential for Embryogenesis Due to Its Influence on Chloroplast Development in Arabidopsis
Source: PLoS One. 2014 Jun 25;9(6):e99741. doi: 10.1371/journal.pone.0099741 (PMC4070914; doi:10.1371/journal.pone.0099741)

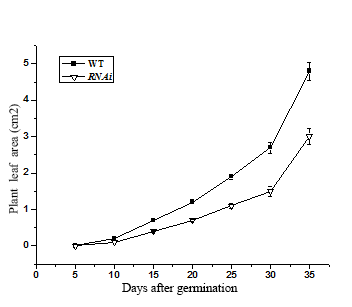

Supplement: Figure S1 — Growth kinetics of the RNAi- Ftshi4 mutant plants. Values are averages ±S.E. of at least six replicated experiments. (TIF) [file pone.0099741.s001.tif]

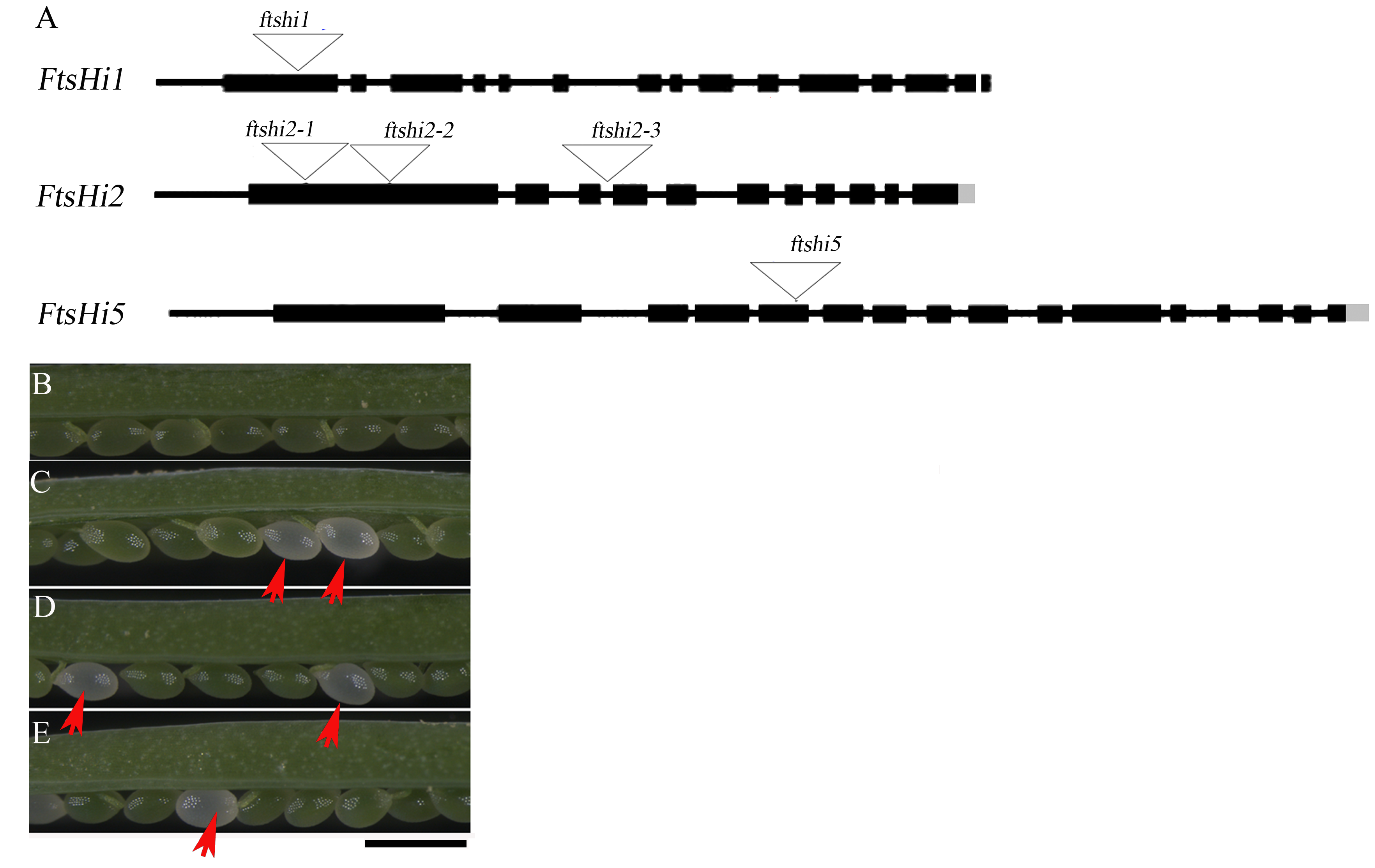

Supplement: Figure S2 — Isolation and characterization of Arabidopsis ftshi1, ftshi2, and ftshi5 mutants. A, Diagram of the T-DNA insertion position in the FtsHi1, FtsHi2, and FtsHi5 genes. Black boxes represent exons. The 5′ untranslated region (UTR) and 3′ UTR are shown in grey boxes. B, 9-DAP dissected wild-type silique. C, 9-DAP dissected ftshi1 (+/−) mutant silique. D, 9-DAP dissected ftshi2 (+/−) mutant silique. E, 9-DAP dissected ftshi5 (+/−) mutant silique. Arrows indicate white ovules with no chlorophyll accumulation. Bar = 1 mm. (TIF) [file pone.0099741.s002.tif]

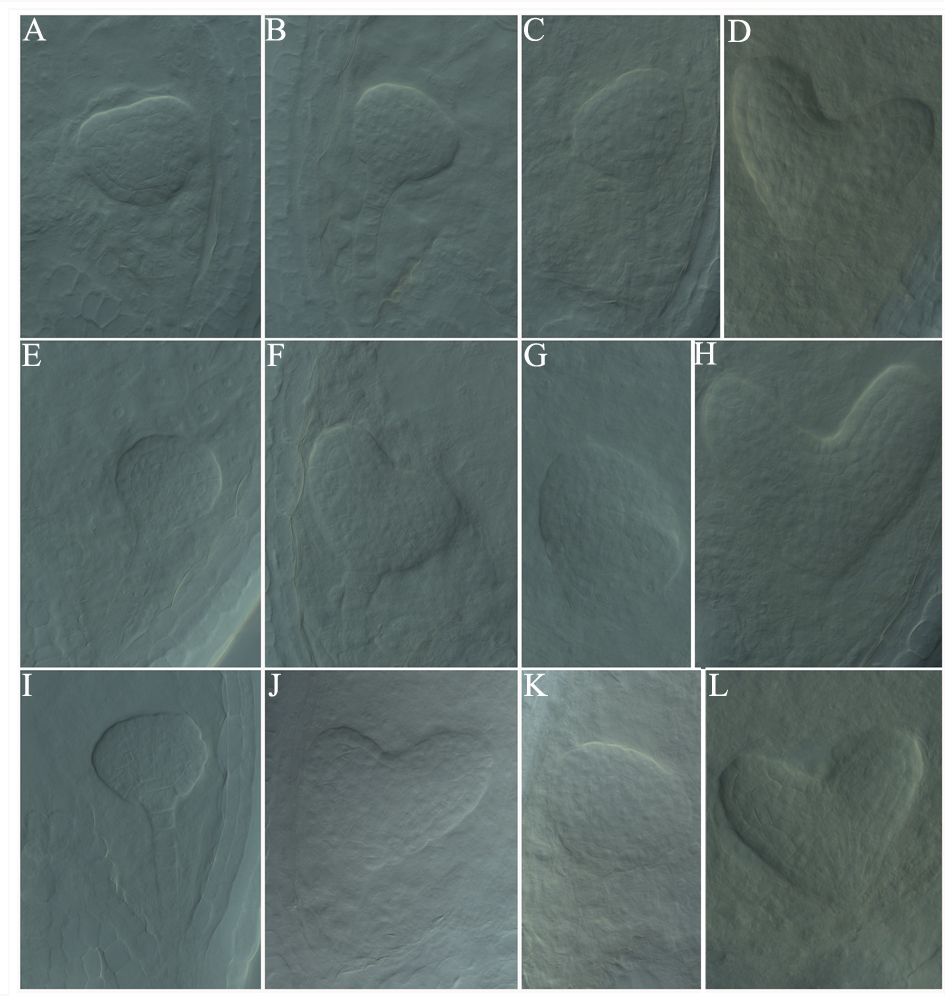

Supplement: Figure S3 — Embryo development of ftshi1 , ftshi2 , and ftshi5 mutants. Mutant embryos from heterozygous ftshi1 plants (A–D), heterozygous ftshi2 plants (E–H), and heterozygous ftshi5 plants (I–L) were retarded and morphologically abnormal compared to wild-type embryos from the same silique. A, E, and I, Mutant embryo development was retarded when wild-type embryo developed to the heart-shaped stage in the same silique. B, F, and J, Mutant embryos showed abnormalities in the regions that developed an embryo axis and radicle compared to wild-type embryos that developed to the early torpedo stage in the same silique. C, G, and K, Mutant globular embryos were morphologically abnormal when wild-type embryos reached maturity in the same silique. D, H, and L, Mutant embryos were arrested at the heart-stage when wild-type embryos reached maturity in the same silique. The wild-type embryo development is the same as in Figure 2. (TIF) [file pone.0099741.s003.tif]

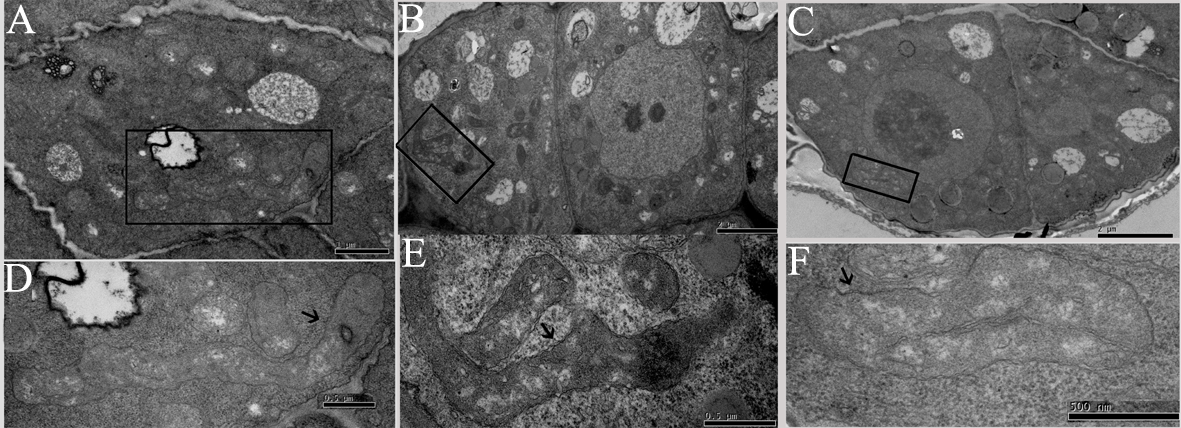

Supplement: Figure S4 — Transmission electron microscopic analysis of plastid development of ftshi1 , ftshi2 , and ftshi5 mutants. A, B, and C, Mutant ftshi1, ftshi2-1, or ftshi5 embryos with development-disrupted “plastids”. Enlargements of the above-described “plastids” are shown in D, E, or F, respectively, and indicated by arrows. Wild-type embryos from each of the same heterozygous ftshi1, ftshi2-1, and ftshi5 siliques are the same as in Figure 3 and therefore not shown. (TIF) [file pone.0099741.s004.tif]

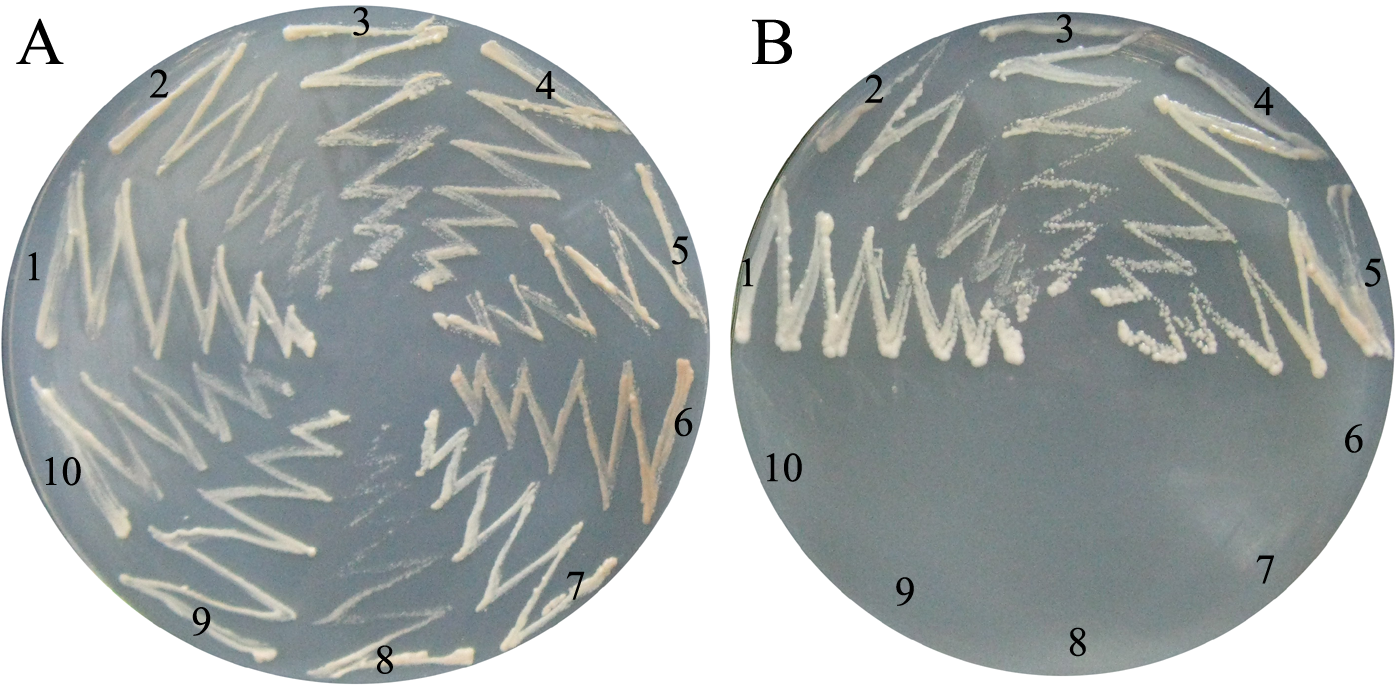

Supplement: Figure S5 — Yeast two-hybrid assay of FtsHi2 and FtsHi4 protein. Yeast cells transformed with the corresponding vectors grew on the -Trp/-Leu medium (A) and -Ade/-His/-Leu/-Trp medium (B). Numbers of 1-10 represent the yeast hybrid with FtsHi proteins as baits or preys, respectively, which is described as following. 1, bait pGBKT7-53 and prey pGADT7-T. 2, bait pGBKT7-FtsHi2 and prey pGADT7-FtsHi2. 3, bait pGBKT7-FtsHi4 and prey pGADT7-FtsHi4. 4, bait pGBKT7-FtsHi2 and prey pGADT7-FtsHi4. 5, bait pGBKT7-FtsHi4 and prey pGADT7-FtsHi2. 6, bait pGBKT7-FtsHi2 and prey pGADT7. 7, bait pGBKT7-FtsHi4 and prey pGADT7. 8, bait pGBKT7 and prey pGBKT7-FtsHi2. 9, bait pGBKT7 and prey pGBKT7-FtsHi4.10, bait pGBKT7-Lam and prey pGADT7-T. (TIF) [file pone.0099741.s005.tif]
