# Supplementary material for: FtsHi4 Is Essential for Embryogenesis Due to Its Influence on Chloroplast Development in Arabidopsis
Source: PLoS One. 2014 Jun 25;9(6):e99741. doi: 10.1371/journal.pone.0099741 (PMC4070914; doi:10.1371/journal.pone.0099741)
Supplement: Table S1 — The interactions between FtsHi proteins in yeast cells. (DOC) [file pone.0099741.s006.doc]

**Table S1.** The interactions between FtsHi proteins in yeast cells

|  | AD | AD-FtsHi1 | AD-FtsHi2 | AD-FtsHi4 | AD-FtsHi5 |
| --- | --- | --- | --- | --- | --- |
| BD |  |  |  |  |  |
| BD-FtsHi1 |  |  |  |  |  |
| BD-FtsHi2 |  |  | **+** | **+** |  |
| BD-FtsHi4 |  |  | **+** | **+** |  |
| BD-FtsHi5 |  |  |  |  |  |

“+” indicates presence of interaction, and “-“ indicates absence of interaction.
