# Supplementary figures and images for: Correction: FtsHi4 Is Essential for Embryogenesis Due to Its Influence on Chloroplast Development in Arabidopsis
Source: PLoS One. 2020 Feb 12;15(2):e0229232. doi: 10.1371/journal.pone.0229232 (PMC7015396; doi:10.1371/journal.pone.0229232)

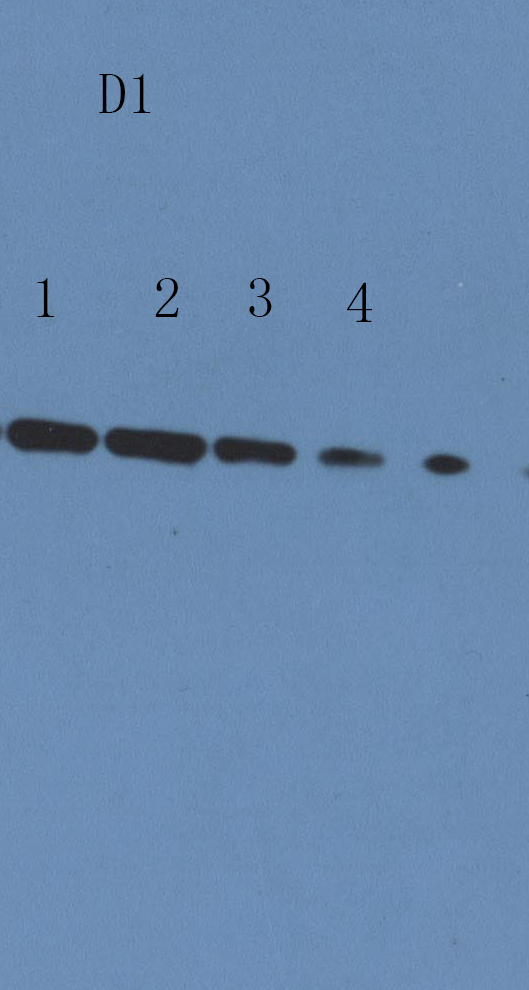

Supplement: S1 File — (TIF) [file pone.0229232.s001.tif]

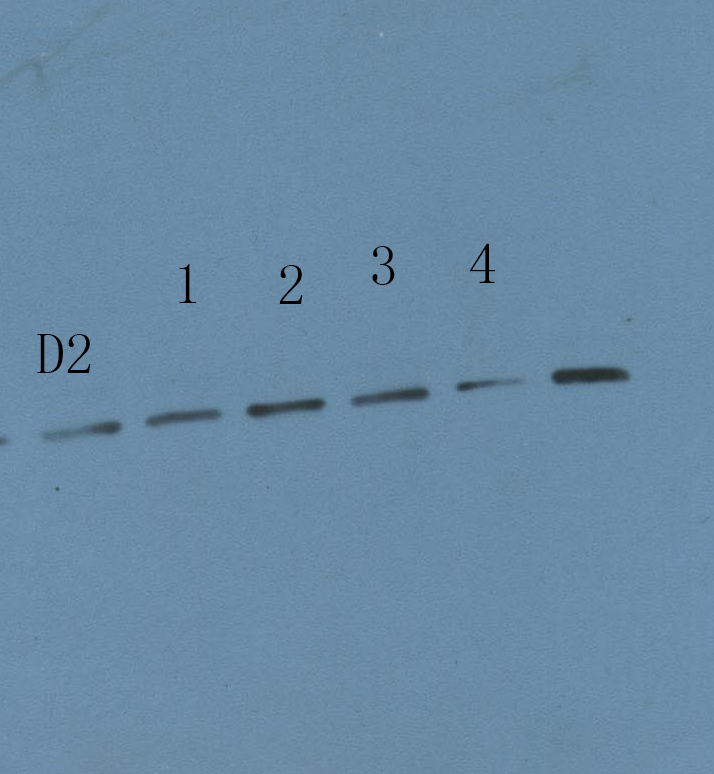

Supplement: S2 File — (TIF) [file pone.0229232.s002.tif]

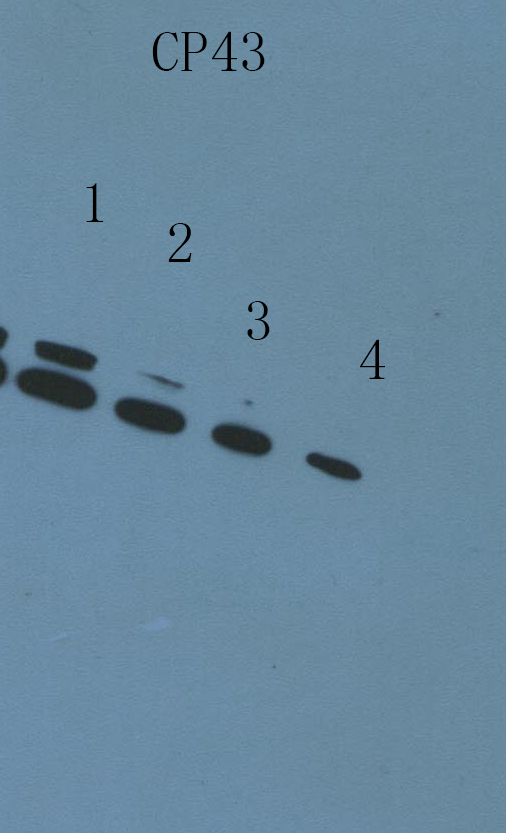

Supplement: S3 File — (TIF) [file pone.0229232.s003.tif]

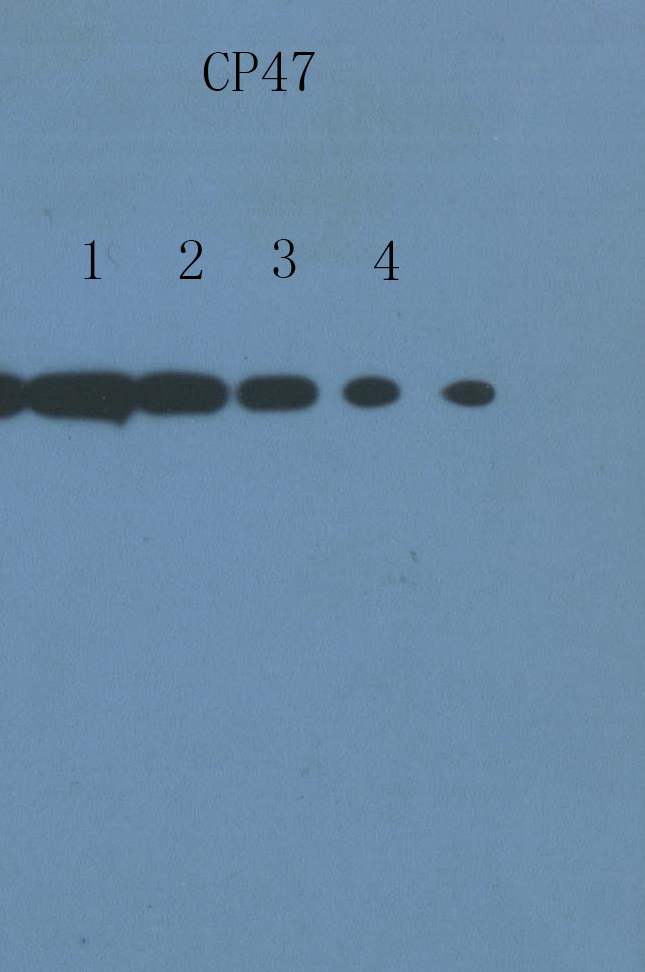

Supplement: S4 File — (TIF) [file pone.0229232.s004.tif]

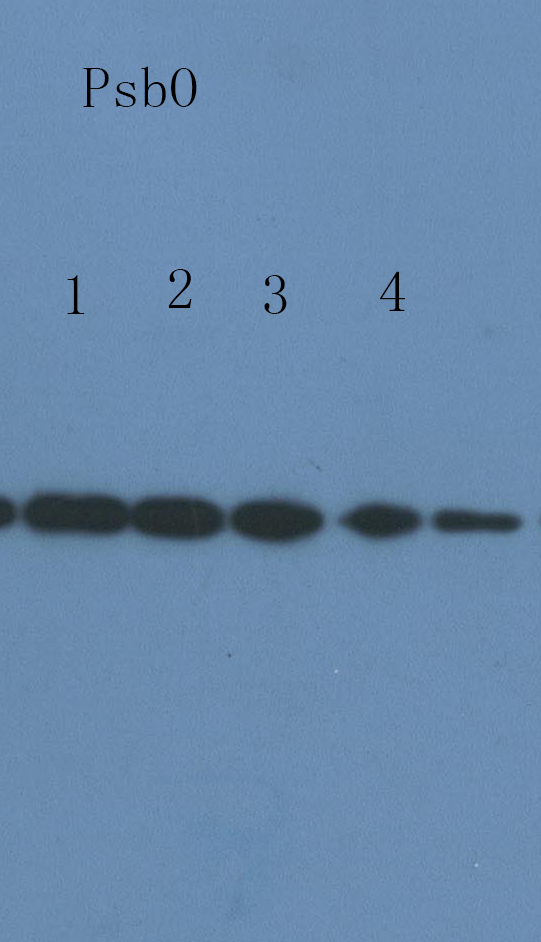

Supplement: S5 File — (TIF) [file pone.0229232.s005.tif]

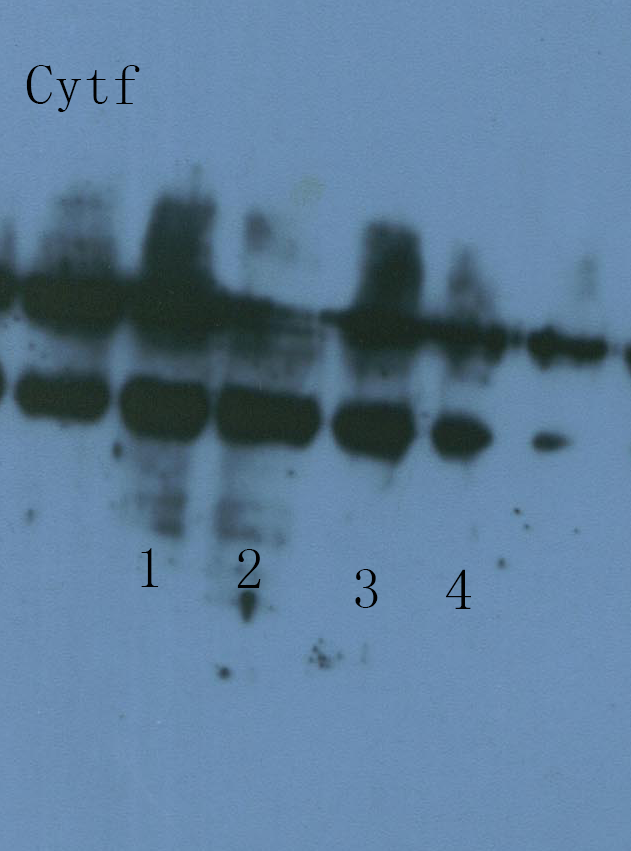

Supplement: S6 File — (TIF) [file pone.0229232.s006.tif]
